# Surface-designed AuNPs-based fluorescent probe for ultra-sensitive detection of oral poultry antibacterial drug furaltadone *via* intermolecular hydrogen bonding†‡

**DOI:** 10.1039/d4ra04293j

**Published:** 2024-09-04

**Authors:** A. Sowndarya, T. Daniel Thangadurai, N. Manjubaashini, M. Pavithrakumar, K. Senthilkumar, D. Nataraj, K. Kadirvelu, K. Naveen Kalagatur

**Affiliations:** a Department of Chemistry and Centre for Research and Development, KPR Institute of Engineering and Technology Coimbatore 641407 Tamilnadu India danielthangadurai.t@kpriet.ac.in; b National Centre for Nanoscience and Nanotechnology, University of Madras Chennai 600025 India; c Department of Physics, Bharathiar University Coimbatore 641046 India; d DRDO-Life Sciences, Bharathiar University Coimbatore 641046 India

## Abstract

Furaltadone (FTD), a nitrofuran drug, was primarily utilized as a very effective oral veterinary antibiotic, especially in poultry production farms. As a result, FTD, a form of carcinogen, might easily enter people *via* the food chain, leading to fatal cancers. As a result, it is critical to develop a quick and efficient approach for detecting FTD at extremely low concentrations. Considering the aforementioned purpose, pamoic acid (PA) capped gold nanoparticles (PA@AuNPs) were synthesized in spherical morphology (size 10–15 nm) using the method of chemical reduction and used as a fluorescent probe to detect FTD. The interaction between PA@AuNPs and FTD was validated by UV-vis, XRD, and FTIR methods. Microscopic images (FESEM and HRTEM) show that PA@AuNPs have varying morphologies including rod, triangle, hexagonal, and pentagonal, and average sizes of 20–50 nm after sensing FTD. The average surface roughness of PA@AuNPs was determined to be 46.75 nm using the AFM technique. The addition of FTD (0 → 100 μM) quenched the fluorescence emission intensity of PA@AuNPs at 436 nm (*λ*_ex_ 353 nm) by 4-fold. This static quenching was confirmed by the formation of a ground state complex, PA@AuNPs·FTD, between AuNPs and FTD using fluorescence lifetime analysis. The presence of an isosbestic point at 412 nm in the UV-visible titration, as well as FTIR data, further demonstrated the existence of this ground state complex. PA@AuNPs revealed high sensitivity (LoD = 9.78 nM; *K*_a_ = 1.0615 × 10^2^ M^−1^) to FTD in water, resulting in a decrease in predicted quantum yield (*Φ*_F_) from 3.36% to 0.35%. To establish PA@AuNPs as a first-generation fluorescence probe for real samples, FTD in blood serum was measured (LoD = 6.07 nM; *K*_a_ = 1.0595 × 10^2^ M^−1^). The non-toxic cytotoxicity and bioimaging in live zebrafish broadened the practical uses of PA@AuNPs. Furthermore, the surface interactions between PA@AuNPs and FTD were studied theoretically using time-dependent density functional theory (TD-DFT) at the B3LYP/6-31G(d,p) level of theory to support the findings from the experiment.

## Abstract 2

Morphology transforming and thermally stable (∼370 °C) PA@AuNPs were utilized for ultra-low detection (LoD 6.0 nM) of oral poultry antibacterial drug Furaltadone in blood serum, supported by DFT studies, and bioimaging in zebrafish.

## Graphical abstract

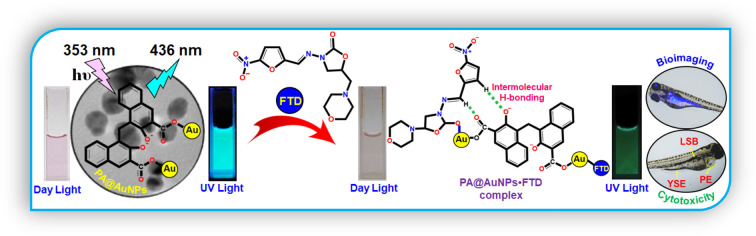

## 1. Introduction

Alarmingly, many nations, including those in the European Union, have outlawed the use of nitrofurans in cattle production due to their carcinogenic effects on humans.^1^ In animal husbandry, poultry refers to domesticated or commercially farmed birds raised for meat, eggs, or feathers.^2^ Bacterial infection, which is unavoidable, affects them as they grow, causing infectious, severe, or persistent illness.^3,4^ To protect the birds, poultry farm owners and farmers administer antimicrobial poultry medications orally.^5^ Furaltadone (FTD), a nitrofuran derivative developed in 1958 by Kefauver, Paberzs, and McNamara,^6^ was used as an oral antibacterial medication to treat and control *Escherichia coli* and *Salmonella* infections in poultry.^7^ However, FTD and its metabolites have been proven to have mutagenic and carcinogenic properties.^8^ As a result, governing bodies such as those of the USA, the European Union (EU), China, and others have banned the use of FTD in meat-producing animals.^9^ The EU's minimal residual concentration limit for FTD and/or its metabolite in edible tissues is 1.0 μg kg^−1^.^10^ Nevertheless, in a few countries, FTD can be administered to cattle, poultry, and aquaculture commodities without a prescription.^11^ Consequently, FTD can readily spread to humans *via* the food chain, causing malignant disorders and even death.^12,13^ As a result, detecting FTD at extremely low concentrations requires a swift and effective approach.

It is noteworthy to note that FTD dissolves in water at a rate of 753 mg L^−1^.^14^ Several reports employ techniques like LC-MS, LC-MS/MS, and so on to detect the presence of FTD.^15^ Chiu *et al.*, Sea-Fue *et al.*, and Shafi *et al.* have previously used electrochemical techniques to detect FTD.^16–18^ These methods are reliable and conclusive, but not all laboratories may have access to such expensive equipment. As a result, when looking for a rapid, selective, sensitive, and dependable tool to determine FTD in an aqueous media, fluorescence may be a viable alternative.^19^ There are currently no literature reports on FTD detection using the fluorescence technique. To use the fluorescence approach to determine FTD, a researcher must carefully design and synthesize a sensor material with suitable photophysical properties.^20^ Based on our previous successful journey and the significance and distinctive properties of AuNPs, such as nonlinear optical, colorimetry, and conductivity,^21^ we fashioned a sensor material that contains a binding site for FTD and exhibits remarkable changes in its photophysical properties upon FTD binding. The optical properties of AuNPs, which result from an enormous electromagnetic field near the surface, are among the most intriguing of these properties, allowing them to be widely used in analytical science, including colorimetric assays, surface-enhanced Raman spectroscopy, surface plasmon resonance (SPR) spectroscopy, and bioimaging.^22^ As previously recorded, the aforementioned unique features of AuNPs are easily accessible due to their variable size, shape, and the surrounding chemical environment.^23,24^ Interestingly, this alteration in the surrounding environment, or surface functionalization of AuNPs with chemical or biological fluorescent ligands, is beneficial for the selective and sensitive detection of an analyte of interest.^25^ Furthermore, surface functionalization is particularly useful for bioimaging the target analyte in living cells^26^ and conducting other biological research.

In light of the aforementioned viewpoint, we chose to functionalize the surface of the AuNPs with pamoic acid (PA) as a fluorescent molecule. Notably, PA served a dual purpose in the current study, operating as both a capping agent (rather than a reducing agent) and a fluorescent component. In pharmacology, PA is commonly employed to prepare repository drug derivatives.^27^ Particularly, the presence of many oxygen atoms in PA allows for significant hydrogen bonding, increasing the drug's solubility in water.^28^ To our astonishment, only two previous studies on PA@AuNPs are now available.^29,30^ The first one determines the ketoconazole (KCZ) using a carbon paste electrode modified with AuNPs, while the second one uses a glassy carbon electrode (GCE) modified with AuNPs. Based on the above statements, none of the two articles has demonstrated the detection of FTD, to the best of our knowledge.

In this communication, we report PA surface functionalized AuNPs of 10–15 nm size with spherical morphology for the detection of FTD, an oral poultry antibacterial medication, in blood serum and bioimaging in live zebrafish. The UV-visible and fluorescence results show PA@AuNPs' ultra-sensitivity to FTD. The changes in the morphology of PA@AuNPs with FTD sensing and their viability for real-sample analysis are also reported. Furthermore, we describe the cytotoxicity of PA@AuNPs against zebrafish embryos. In addition, the PA@AuNPs were successfully employed in the fluorescence bioimaging of FTD in zebrafish. TD-DFT at the B3LYP/6-31G(d,p) level of theory research provides strong support for the current experimental results. As far as we know, this is the first report on PA@AuNPs being utilized to detect FTD by the fluorescence spectroscopic approach.

## 2. Experimental section

AuNPs' surfaces can be functionalized with appropriate functional moieties, making them useful in specialized applications such as selective analyte detection.^30^ As previously stated, despite being a fluorescent molecule, PA can operate as both a reducing and capping reagent.^31,32^ However, in the current investigation, PA was used as a capping agent rather than a reducing agent to produce PA@AuNPs with a spherical shape. The PA@AuNPs were synthesized by literature method^33^ The purity, crystalline nature, chemical composition, binding energy, shape, functional groups, optoelectronic capabilities, and thermal stability of PA@AuNPs were all determined using standard physicochemical characterization techniques.

### 2.1. Materials and methods

FTD was acquired from Sigma Aldrich and used without additional purification. All experimental solutions were made with deionized (DI) water obtained from a water purification system (AQUOION TBD 200, Two Bed Demineraliser).

The UV-visible spectra were obtained with a UV-1900 spectrophotometer (Model SHIMADZU). Powder X-ray diffraction (XRD) patterns were acquired using a Bruker D8 Advance X-ray diffractometer, using Ni-filtered Cu-Kα radiation (*λ* = 1.5418 Å). Selectivity and interference studies were measured by using a JASCO FP-8250 spectrofluorometer. Fourier transform infrared (FT-IR) spectra were captured using an IR Affinity – 1S (MIRacle – 10) at the Avinashilingam Institute for Home Science and Higher Education for Women in Coimbatore, India. Raman analysis was carried out at SAIF, MGU, Kottayam, India, using a LabRAM-HR Raman spectrometer (Horiba) and a 532 nm excitation laser source. The structural morphology was investigated by FESEM (ZEISS (SIGMA), BRUDER) at Coimbatore Institute of Technology, Coimbatore, India, and HRTEM (200 kV FE-TEM, model JEM-2100F, JEOL) at MGU, Kottayam, India. AFM pictures were captured on an Alpha300RA AFM and RAMAN model at SAIF, MGU, Kottayam, India. The fluorescence spectra were obtained using a JASCO FP-6500 spectrofluorometer with a Xenon discharge lamp at GRI in Dindigul, India, and a PerkinElmer fluorescence spectrometer FL 6500 at IIT Palakkad in Kerala, India. The PL lifetime measurements were performed using an IBH time-correlated single photon counting (TCSPC) system, and the decay profile was deconvoluted using IBH data station software V2.6 at Bharathiar University in Coimbatore, India. The cytotoxicity and fluorescence bioimaging investigations were carried out using an EVOS Life Technology Spectro fluorophotometer at the DRDO-Life Science Centre, Bharathiar University, Coimbatore, India. The Department of Physics at Bharathiar University in Coimbatore, India, conducted computational research utilizing density functional theory at the B3LYP1/6-311G(d,p) level of theory.

### 2.2. Synthesis of PA@AuNPs

PA@AuNPs were synthesized as per the literature procedure.^34^ Briefly, under sonication, 140 μL of 3.0 M NaOH solution was added to 10 mL of PA solution to prepare a clear solution of the disodium salt of pamoic acid (Na_2_-PA) (6.0 mM). After adding 10 mL of HAuCl_4_·3H_2_O to the Na_2_-PA solution, the sonication was continued for one hour. To complete the growth of PA@AuNPs, the reaction mixture was stored at room temperature for one day. This solution was then washed with distilled water 30 mL, centrifuged, and dried in a hot air oven for 6 hours (100 °C).

### 2.3. Fluorescence studies

Fluorescence analysis was used to determine the FTD using PA@AuNPs. Fluorescence emission intensity changes were measured during titration experiments when FTD was added to the aqueous solution of PA@AuNPs. A 353 nm excitation (*λ*_ex_) wavelength was used for all measurements, and where appropriate, 5 nm slit widths were used for both excitation and emission. Each titration experiment was repeated at least thrice to ensure consistent results. The quenching rate constant (*K*_q_) was calculated using the Stern–Volmer equation, and the association constant (*K*_ass_) was determined using the Scatchard plot.

### 2.4. Binding constant and LoD values calculations

The binding strength and limit of detection (LoD)^35^ between PA@AuNPs and FTD were computed by using the following equation.

1 log[*F* − *F*_0_/*F*_0_] = log *K*_a_ + *n* log[*Q*]

where “*F*” is the fluorescence intensity after the addition of FTD and “*F*_0_” is the initial fluorescence intensity of PA@AuNPs. “*K*_a_ is the binding constant, “*n*” is the number of binding sites, and “*Q*” is the concentration of FTD. The *K*_a_ and ‘*n*’ can be estimated from the intercept and slope obtained by plotting log[*F* − *F*_0_/*F*_0_] against log[*Q*]. The fluorescence intensity was measured with the addition of FTD in different concentrations [*Q*] to the solution of PA@AuNPs and also in the blood serum sample. LoD was obtained from the slope and intercept (eqn (2)).

2 LoD = [3 × (intercept/slope)]

### 2.5. Bioimaging studies

To investigate the biological uses of PA@AuNPs, the cytotoxicity of PA@AuNPs was initially assessed using zebrafish larvae. AB strain zebrafish were reared following OECD (Organisation for Economic Co-operation and Development) recommendations (OECD, 2013) at 28 °C with a 14 h light and 10 h dark cycle. Spawning was induced with 6 male and 12 female zebrafish (1 : 2 ratio), following OECD 236 standards. The eggs were first gathered in sunlight, and unfertilized eggs were removed with an inverted microscope (EVOS FLC, Life Technologies). Fertilized eggs were used to evaluate the toxic effects and bioimaging of PA@AuNPs.^36^

## 3. Results and discussion

### 3.1. Estimation of selectivity and sensitivity

**Fig. 1:**
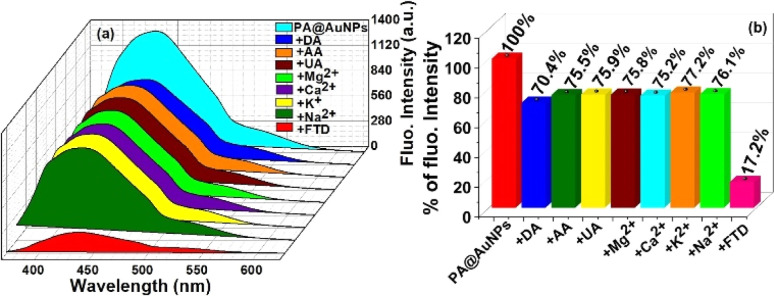
(a) 3D diagram of fluorescence emission intensity changes of PA@AuNPs (1.0 mM) upon addition of various analytes (100 μM) (*λ*_ex_ 353 nm; slit width 5 nm), and (b) corresponding bar diagram shows the emission intensity of quenching percentage (STD = 3%).

To examine the selectivity of FTD at PA@AuNPs, we have carried out the fluorescence experiment in the presence of various analytes, including FTD, dopamine (DA), ascorbic acid (AA), uric acid (UA), magnesium ions (Mg^2+^), calcium ions (Ca^2+^), potassium ions (K^+^), and sodium ions (Na^+^) (Fig. 1). The addition of FTD (100 μM) reduced the fluorescence emission intensity (∼6-fold) of PA@AuNPs (1.0 mM) and resulted in a redshift (436 nm → 439 nm; 3 nm; *λ*_ex_ 353 nm) compared to other analytes. This fluorescence intensity decrease might be due to the formation of a ground state complex between FTD and PA@AuNPs. *i.e.* static quenching process. The fluorescence quantum yield (*Φ*_F_) of PA@AuNPs (3.36%) was reduced to 0.35% upon the addition of FTD. The addition of other analytes (Na^+^, K^+^, Ca^2+^, Mg^2+^, GLU, DA, and AA) did not significantly alter the fluorescence emission under similar experimental conditions, indicating that there was either no binding or very weak binding between these analytes and PA@AuNPs. These results demonstrated that PA@AuNPs are highly selective for FTD sensing. The corresponding fluorescence spectrum can be found in Fig. SI1.‡

**Fig. 2:**
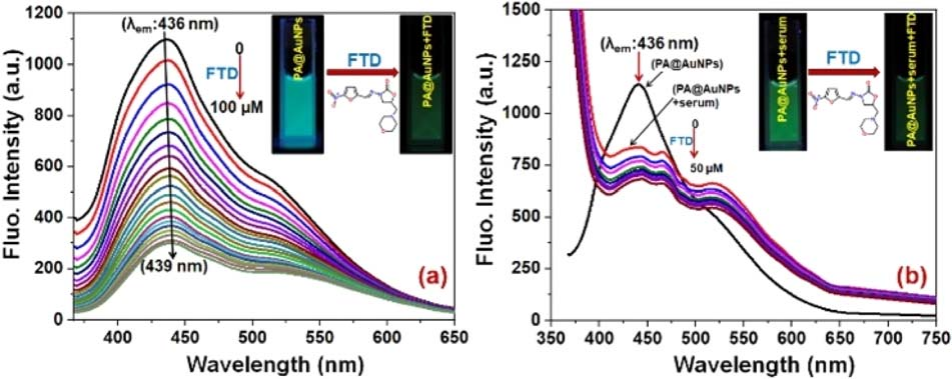
Fluorescence emission spectrum of PA@AuNPs (1.0 mM) upon increasing the concentration of (a) FTD (0 → 100 μM), and (b) FTD in blood serum (0 → 50 μM) (*λ*_ex_ 353 nm; slit width 5 nm) (insets: corresponding reaction solutions under UV-lamp).

Concerning the selectivity study results, a fluorescence titration experiment was used to assess PA@AuNPs' sensitivity to FTD. The fluorescence intensity of the PA@AuNPs solution (1.0 mM) was promptly quenched by adding varied doses of FTD (0–100 μM). The addition of FTD reduced the fluorescence intensity of PA@AuNPs by 4-fold and caused a red shift (436 → 439 nm; 3 nm) (Fig. 2a). The reduction in fluorophore (PA@AuNPs) emission intensity is induced by a wide range of molecular interactions, including energy transfers, excited-state reactions, ground-state complex formation, and collisions.^37^ In this study, static quenching occurs due to the development of a ground state complex between the quencher (FTD) and fluorophore (PA@AuNPs), as validated by FTIR and UV-visible measurements. The fluorescence titration investigations yielded binding constant (*K*_a_) and LoD values of 1.0615 × 10^2^ M^−1^ and 9.78 nM (*R*^2^ = 0.991; *n* = 3%), respectively (Fig. SI2‡). Interestingly, the fluorescent probe, PA@AuNPs, had the lowest detection limit while simultaneously demonstrating good analytical performance in real sample analysis. To demonstrate PA@AuNPs' real-world feasibility, FTD was detected in a blood serum sample. When blood serum was added to the PA@AuNPs solution, the fluorescence intensity was reduced. Adding varied concentrations of FTD (0–50 μM) to PA@AuNPs with blood serum lowered their fluorescence intensity further (Fig. 2b). The fluorescence titration investigation (Fig. SI3‡) shows that PA@AuNPs can detect FTD in blood serum samples with a LoD of 6.07 nM and *K*_a_ of 1.0595 × 10^2^ M^−1^ (*R*^2^ = 0.995; *n* = 3%), indicating their potential for practical applications.

### 3.2. Time-correlated single photon counting (TCSPC)

**Fig. 3:**
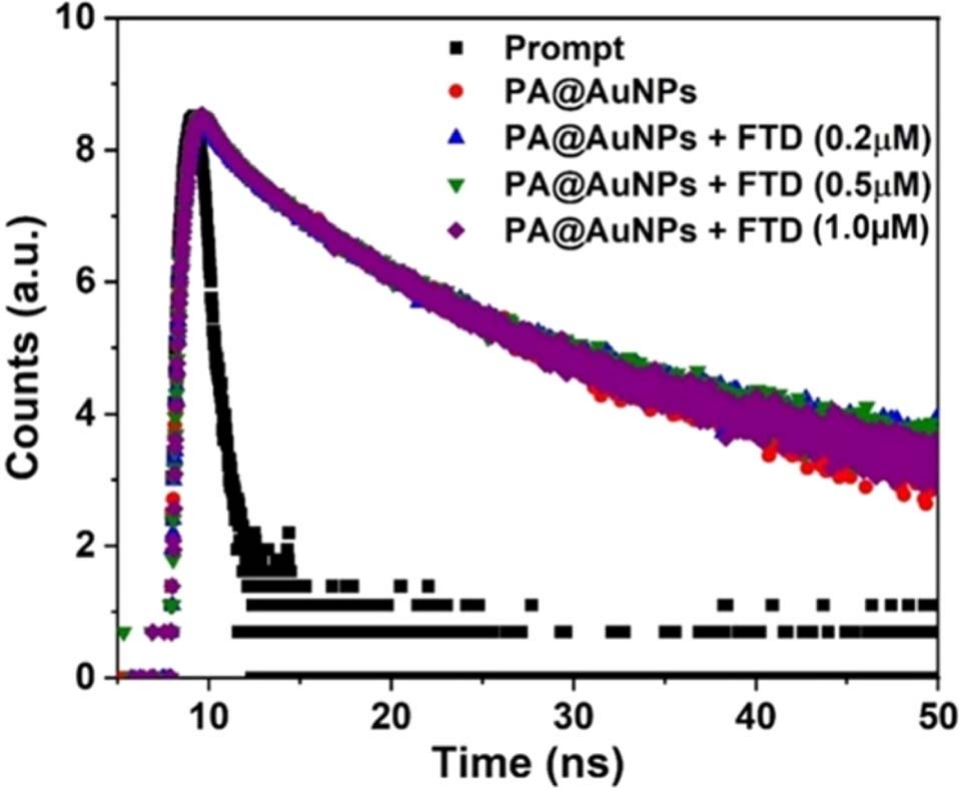
Fluorescence decay profile of PA@AuNPs (1.0 mM) in the absence and presence of FTD (0.2, 0.50, and 1.0 μM, respectively) (*λ*_ex_ = 450 nm).

To further understand the fluorescence quenching behavior of PA@AuNPs after the addition of FTD, Time-Correlated Single Photon Counting (TCSPC) was performed on PA@AuNPs with and without FTD at an excitation wavelength of 450 nm. In the absence of FTD, the fluorescence lifetime profile of PA@AuNPs (1.0 mM) is best suited with a tri-exponential function, resulting in an average lifetime of 10.04 ns. This tri-exponential decay data shows the presence of three different species, including free and FTD-bound forms of PA@AuNPs. Adding varied doses of FTD (0.25, 0.5, and 1.0 μM) to PA@AuNPs resulted in fluorescence lifetime values of 9.07, 6.85, and 8.72 ns, respectively (Fig. 3; Table 1). The TCSPC results indicate a decrease in the decay time of PA@AuNPs at the highest concentration of FTD (1.0 μM) due to the formation of a non-fluorescent ground state complex, PA@AuNPs·FTD. These findings also verified the static quenching mechanism, which involves the formation of a ground state complex between the quencher (FTD) and fluorophore (PA@AuNPs).^38^ Furthermore, it is clear that in static quenching, fluorescent molecules, and quenchers combine to create non-fluorescent complexes.^39^ The difference in fluorescence lifetime between PA@AuNPs with and without FTD (0.25 → 0.5 → 1 μM) was 0.97, 2.22, and 1.87 ns. This lifetime difference verifies the static quenching mechanism of PA@AuNPs after the addition of FTD, which is consistent with fluorescence titration data.

**Table 1:** Relative fluorescence and average fluorescence lifetime of PA@AuNPs (1.0 mM) upon addition of FTD (0.25, 0.50, and 1.0 μM) in H_2_O (*λ*_ex_ = 450 nm)

Sample name	*τ* values (ns)	Relative amplitude	CHISQ	Average lifetime value (ns)
PA@AuNPs (tri-exponential)	*τ*_1_ – 8.94	*B*_1_ – 15.35	1.08	10.04
	*τ*_2_ – 4.59	*B*_2_ – 63.76		
	*τ*_3_ – 1.53	*B*_3_ – 20.89		
PA@AuNPs (1 mL) + 0.25 μM FTD (1 mL)	*τ*_1_ – 3.61	*B*_1_ – 50.82	0.94	9.07
	*τ*_2_ – 6.03	*B*_2_ – 12.00		
	*τ*_3_ – 1.55	*B*_3_ – 37.18		
PA@AuNPs (1 mL) + 0.5 μM FTD (1 mL)	*τ*_1_ – 3.72	*B*_1_ – 52.05	1.02	6.85
	*τ*_2_ – 6.07	*B*_2_ – 12.23		
	*τ*_3_ – 1.17	*B*_3_ – 35.72		
PA@AuNPs (1 mL) + 1.0 μM FTD (1 mL)	*τ*_1_ – 3.83	*B*_1_ – 53.95	1.00	8.72
	*τ*_2_ – 6.18	*B*_2_ – 13.04		
	*τ*_3_ – 1.15	*B*_3_ – 33.01		

### 3.3. Interference studies with different interfering compounds and time effect

Interference studies are vital in demonstrating the selectivity of FTD employing PA@AuNPs. This fluorescence study was conducted in the presence of FTD and other interferences such as PA, AZ, EZ, UA, AA, DA, GLU, Na^+^, Ca^2+^, and Mg^2+^, (Fig. SI5‡). There is evidence that FTD responds quickly with a drop in fluorescence intensity of PA@AuNPs, even when the number of common interferents increases 100-fold. As a result, we confirm that the other common species does not interfere with FTD, and FTD is strongly bonded with PA@AuNPs.

A fluorescence analysis of FTD sensing by PA@AuNPs was undertaken to investigate the effect of time. Sensing experiments were conducted utilizing the FTD (100 μM) at different time intervals (*λ*_ex_ 353 nm). The addition of FTD to PA@AuNPs (1.0 mM) caused a dramatic drop in fluorescence intensity within a minute (*λ*_em_ 436 nm). PA@AuNPs showed no maximum intensity alterations even after 30 minutes of exposure to FTD (Fig. SI5‡). This data implies that PA@AuNPs have good sensing behavior for FTD and can detect it within a minute.

### 3.4. Reversibility test with EDTA and effect of pH

The reversible fluorescence “off–on” property of PA@AuNPs was examined with FTD and a chelating agent namely, ethylenediamine tetra acidic acid (EDTA). These solutions were alternatively added to the PA@AuNPs solution. Fig. SI7‡ demonstrated that the fluorescence intensity of PA@AuNPs was decreased by adding FTD (100 μM) and increasing after the addition of EDTA (100 μM). This cycle was carried out repeatedly for three times. This reversible study demonstrated that PA@AuNPs show a strong bond with FTD even in the presence of EDTA.

We conducted the FTD sensing performance of PA@AuNPs under different pH conditions to further examine the detection capability of the sensor probe. Initially, the PA@AuNPs and PA@AuNPs·FTD solution have neutral pH 7.0. The fluorescence changes across various pH ranges from 1.0 to 13.0 were displayed in Fig. SI8 (acidic) and SI9‡ (basic). In the acidic condition (pH 1.0, 3.0, and 5.0), the protonation may have caused a change in the fluorescence intensity (Fig. SI8‡). In contrast, the fluorescence intensity varies with blue shift (439 → 431 nm; 9 nm) under the basic conditions (pH 9.0, 11.0, and 13.0) (Fig. SI9‡). This might be due to the interaction between the hydroxide ion and PA@AuNPs·FTD complex. However, pH 7 was found to be ideal for the fluorescence signal response of PA@AuNPs for FTD detection. This outcome indicates that PA@AuNPs have the potential tool to be used in biological applications.

### 3.5. Investigation of structural and morphological changes of PA@AuNPs after the interaction with FTD

#### 3.5.1. X-ray diffraction analysis of PA@AuNPs·FTD

**Fig. 4:**
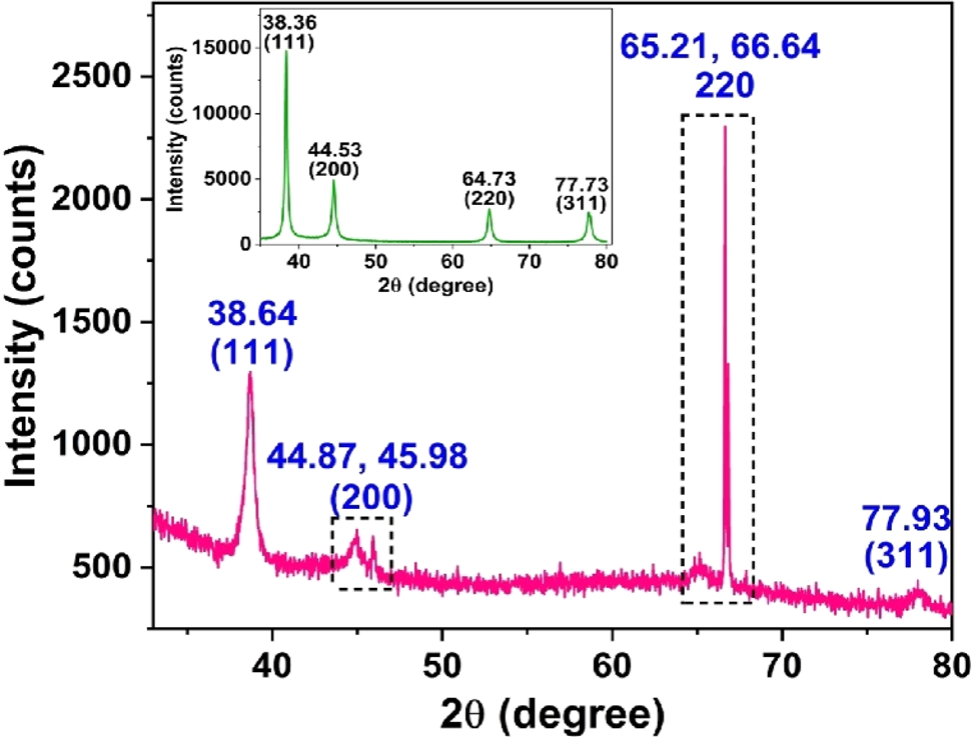
XRD pattern of PA@AuNPs·FTD (inset: XRD of PA@AuNPs).

To investigate the crystalline nature and structure of PA@AuNPs·FTD, we conducted an XRD analysis. PA@AuNPs·FTD exhibited strong and high-intensity diffraction peaks at 2*θ* 38.64°, 44.87°, 45.98°, 65.21°, 66.64°, and 77.93°, corresponding to the (111), (200), (220), and (311) planes. A comparison of XRD of PA@AuNPs and PA@AuNPs·FTD shows that AuNPs in PA@AuNPs·FTD are pure and crystalline, while PA@AuNPs·FTD is less intense (Fig. 4). Interestingly, the intensity of the (220) plane of PA@AuNPs·FTD is 2-fold higher than that of other planes, and the (200) and (220) planes of PA@AuNPs·FTD are generated by splitting due to closely-spaced stacking of the complex that formed with morphological changes. These morphological changes could be confirmed by microscopy analyses.

#### 3.5.2. HRTEM, FESEM and AFM analysis of PA@AuNPs·FTD

**Fig. 5:**
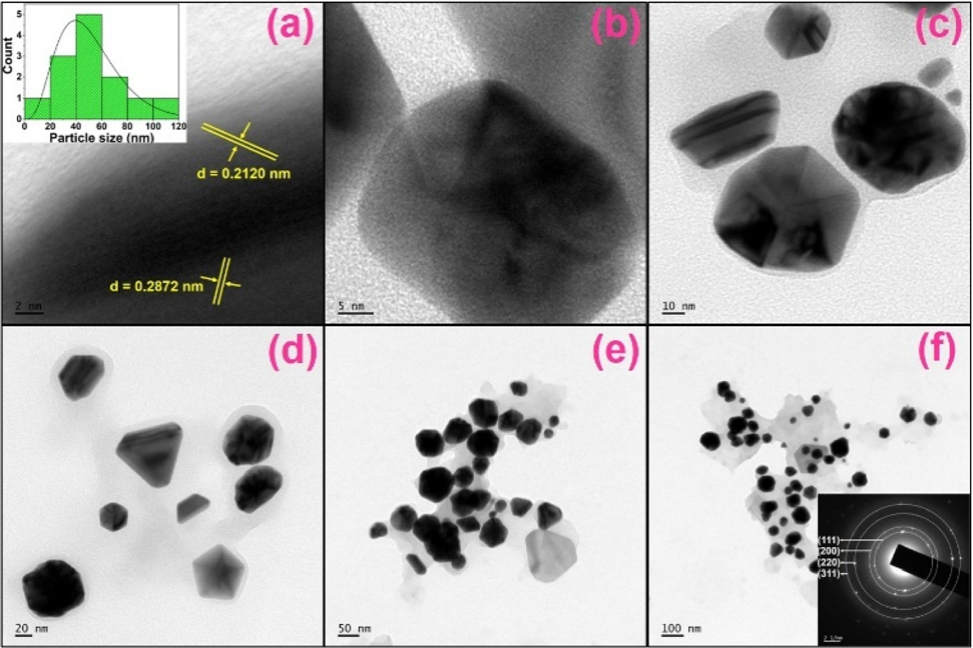
HRTEM images of PA@AuNPs·FTD in a different scale (a → f; 2, 5, 10, 20, 50, and 100 nm), (inset a) histogram of PA@AuNPs of the average diameter of 20–50 nm, and (inset f) SAED pattern of PA@AuNPs·FTD. The HRTEM images of PA@AuNPs can be found in Fig. SI10.‡

**Fig. 6:**
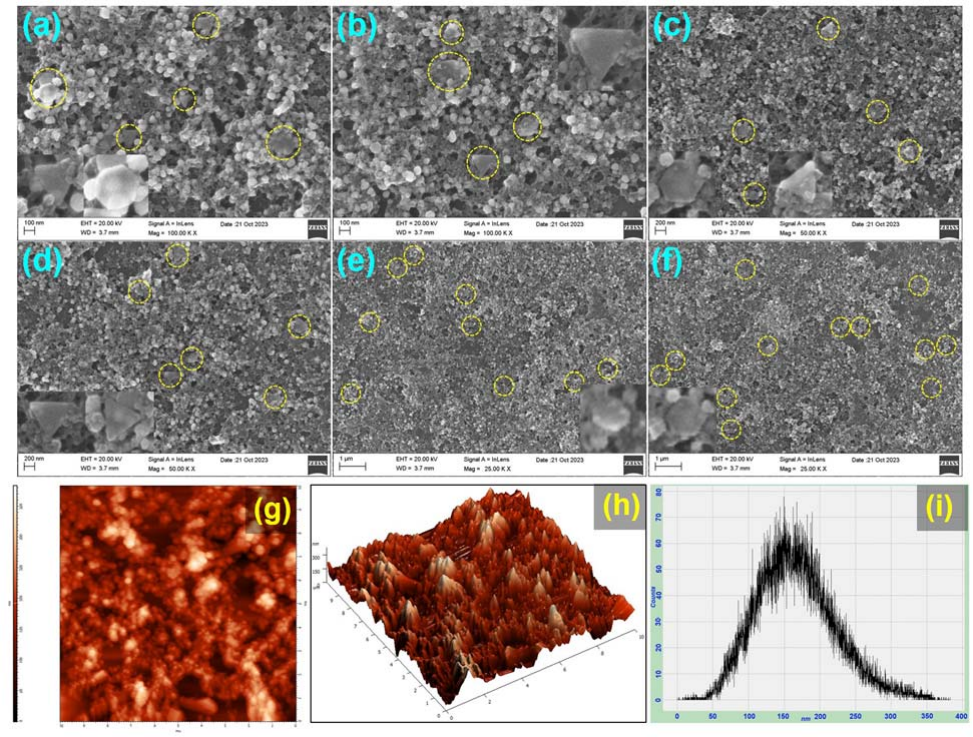
(a–f) FESEM images of PA@AuNPs·FTD (a) 100 nm, (b) 100 nm, (c) 200 nm scale, (d) 200 nm, (e) 1 μm, and (f) 1 μm, respectively, and (g–i) AFM images of PA@AuNPs·FTD (g) 2D mode, (h) 3D mode, and (i) high profile graph. The FESEM and AFM images of PA@AuNPs can be found in Fig. SI11.‡

The morphology of PA@AuNPs·FTD was studied using HRTEM and FESEM techniques. The HRTEM images of PA@AuNPs·FTD show various morphologies, including rod, triangle, hexagonal, and pentagonal, with an average size of 20–50 nm (Fig. 5a inset). High-resolution TEM images revealed two different types of fringes with an estimated average *d*-spacing value of 0.2496 nm (Fig. 5a). According to the SAED pattern (Fig. 5f inset), PA@AuNPs exhibit a good crystalline structure. The crystals are structured in distinct directions, which complements the XRD pattern. The morphology of PA@AuNPs·FTD is a triangle and hexagonal shape, as supported by FESEM images (Fig. 6a–f). It is noteworthy to emphasize that HRTEM and FESEM images of the PA@AuNPs confirmed the spherical shape and average size of 10–15 nm (Fig. SI10 and SI11A‡). Interestingly, one of our previous studies elucidated that the spherical morphology of PA@AuNPs was changed to octahedral when they interacted with Levofloxacin (LF), an antibacterial drug.^34^ The EDAX findings confirm that all elements are present in PA@AuNPs·FTD, with the chemical composition of Au 3.00, C 17.73, O 78.50, and F 0.78% (Fig. SI12; Table SI1‡). The elemental fluorine (0.78%) detected in PA@AuNPs·FTD could be attributed to impurity on the surface during the sensing and/or characterization procedure. We took AFM images in 2D and 3D modes to investigate the surface topology of PA@AuNPs·FTD (Fig. 6g–i). The average roughness (*S*_a_) determined *via* AFM analysis is found to be 46.75 nm. This average roughness of PA@AuNPs·FTD was higher than that of bare PA@AuNPs (*S*_a_ = 20.72 nm) (Fig. SI11B‡) which further supports the presence of FTD on PA@AuNPs surface.

#### 3.5.3. UV-visible spectrum of PA@AuNPs·FTD

Absorption titration experiments were conducted to investigate the interaction between PA@AuNPs and FTD. The UV-visible spectrum of FTD (1.0 mM) shows a significant absorption band at 260 (π → π* transition) and 365 nm (n → π* transition), respectively (Fig. SI10‡ inset). The addition of 10 μL of FTD to PA@AuNPs solution caused immediate changes in absorption intensity, including a red-shift (546 → 562 nm; 16 nm) of the AuNPs absorption band and the formation of an isosbestic point at 412 nm (Fig. SI10‡). Adding FTD (0 → 100 μL of 1.0 mM) to the PA@AuNPs solution (1.0 mM) decreased absorption intensity and raised FTD (365 nm; n → π*) significantly. The changes in absorption intensity and red-shift strongly point to a static interaction between PA@AuNPs and FTD, which results in the development of a ground state complex, PA@AuNPs·FTD. UV-visible absorption measurements revealed a limit of detection (LoD) of 13.0 nM and a binding constant of 7.55 M^−1^ (*R*^2^ = 0.983) (Fig. SI11‡).

#### 3.5.4. FTIR spectrum of PA@AuNPs·FTD

**Fig. 7:**
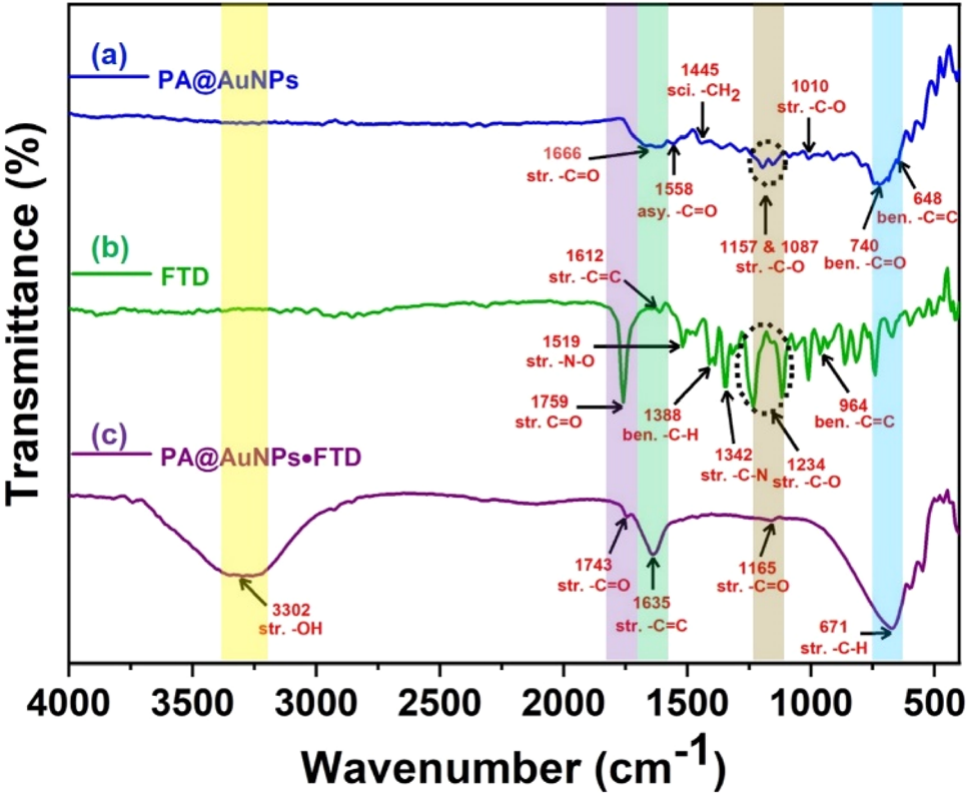
FT-IR spectrum of PA@AuNPs (a), FTD (b), and PA@AuNPs·FTD (c).

The FTIR spectrum provided additional evidence for the formation of the PA@AuNPs·FTD complex (Fig. 7). The stretching and the asymmetric stretching vibrations of –C=O in PA@AuNPs were observed at 1666 and 1558 cm^−1^. The peaks at 1445, 1157, 1087, and 1010 cm^−1^ correspond to the scissoring vibration of –CH_2_, and the possible vibration of the –C–O bond (Fig. 7a). FTD's FTIR spectra revealed a stretching vibration of –C=O at 1759 cm^−1^, as well as asymmetric and bending vibrations of –C=C– at 1612 and 964 cm^−1^, respectively. The stretching vibrations of –N–O and –C–N were detected at 1519 and 1342 cm^−1^, respectively. The bands seen at 1388 and 1234 cm^−1^ correspond to the bending and stretching vibrations of –C–H and –C–O, respectively (Fig. 7b).^40^ Interestingly, there is no peak was observed for the –OH group in both PA@AuNPs and FTD FTIR spectra. The formation of PA@AuNPs·FTD ground state complex resulted in a new –OH stretching band at 3302 cm^−1^, while peak shifts in the other stretching vibrations of –C=O, –C=C, and –CH were found at 1743, 1165, 1635, and 671 cm^−1^, respectively (Fig. 7c). These FTIR data corroborate the development of the PA@AuNPs·FTD complex, which facilitates UV-visible titration studies.

#### 3.5.5. Zeta potential measurement

To predict the long-term stability of colloidal dispersions, it is essential to understand the state of the gold nanoparticle surface.^41^ The zeta potential value of the bare PA@AuNPs is −18.1 mV, indicating that the synthesized AuNPs are more stable and the obtained negative charge value represents the charge of its surface (Fig. SI15a‡). The zeta potential value of PA@AuNPs was decreased further (−12.5 and −9.12 mV) by the addition of different concentrations of FTD (0.5 and 0.75 μM) (Fig. SI15b and c‡). The charge variations on the surface of AuNPs provide a clear explanation for the chemical reactions observed, and AuNPs have a significant reduction in negative charge, and form a positive surface due to H-bond covering their surfaces.^42^

### 3.6. Bioimaging studies for the detection of FTD in zebrafish embryos

Among the various biological models, the zebrafish (*Danio rerio*) has been deemed the most promising and useful model for practical applications in nanomedicines due to several attributes, including economic rearing (when compared to rodents), operator simplicity, embryo and larvae have small size (0.5–5.0 mm), high fertility (up to hundreds of eggs per day), rapid ex utero development, availability of transgenic lines, and, most remarkably, optical transparency and, most importantly, 90% of the genes are homologous to humans.^43–46^ Furthermore, unlike mice, zebrafish are optically transparent, enabling real-time observation of cell activities such as vasculogenesis and organogenesis.^47^ On average, zebrafish live for three years, and they can live up to five years in laboratory conditions.^48^

**Fig. 8:**
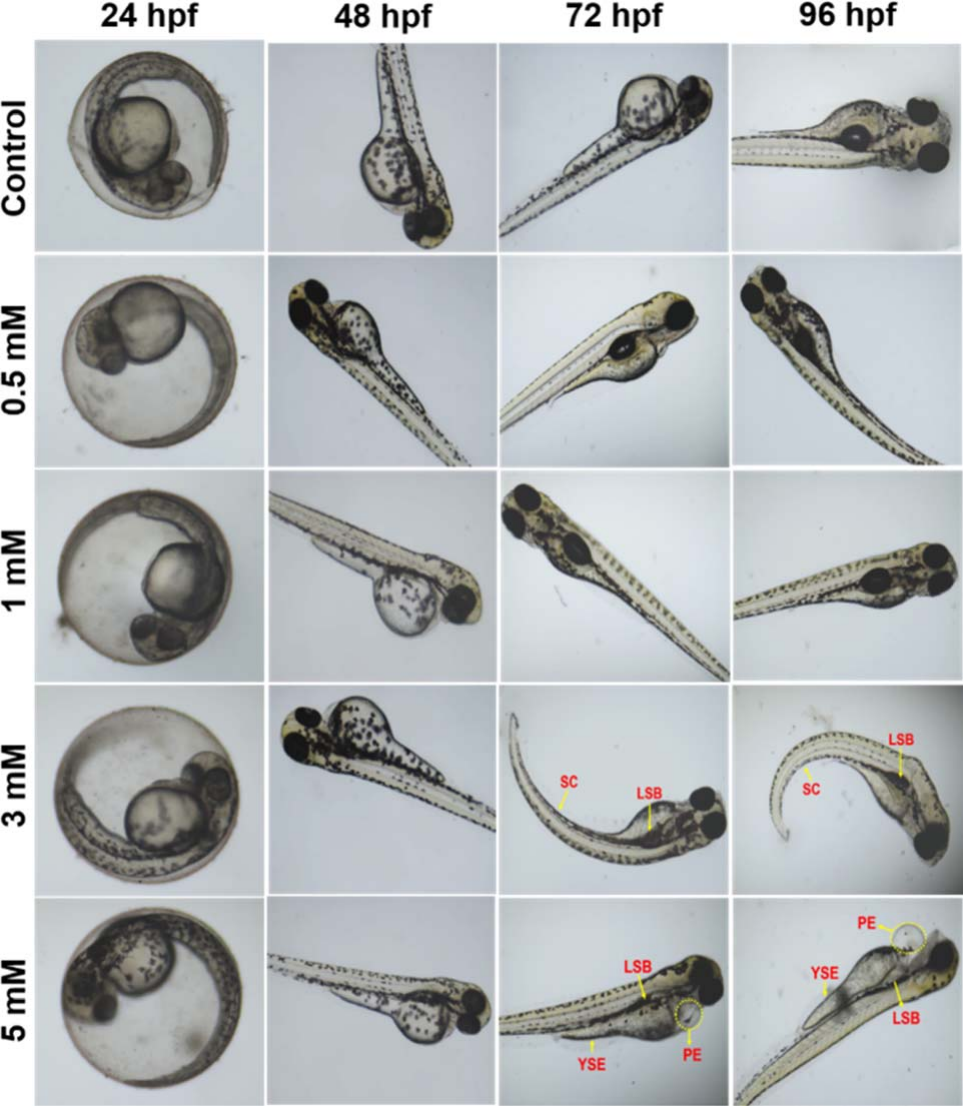
Morphological changes in zebrafish embryos affected by various concentrations of PA@AuNPs (0.5, 1, 3, and 5 mM). The control showed normal development of an embryo, and the PA@AuNPs exposed to the embryo showed deformities like SC-spinal curvature, LSB-lack of the swim bladder, YSE-yolk solk edema, and PE-pericardial edema.

**Fig. 9:**
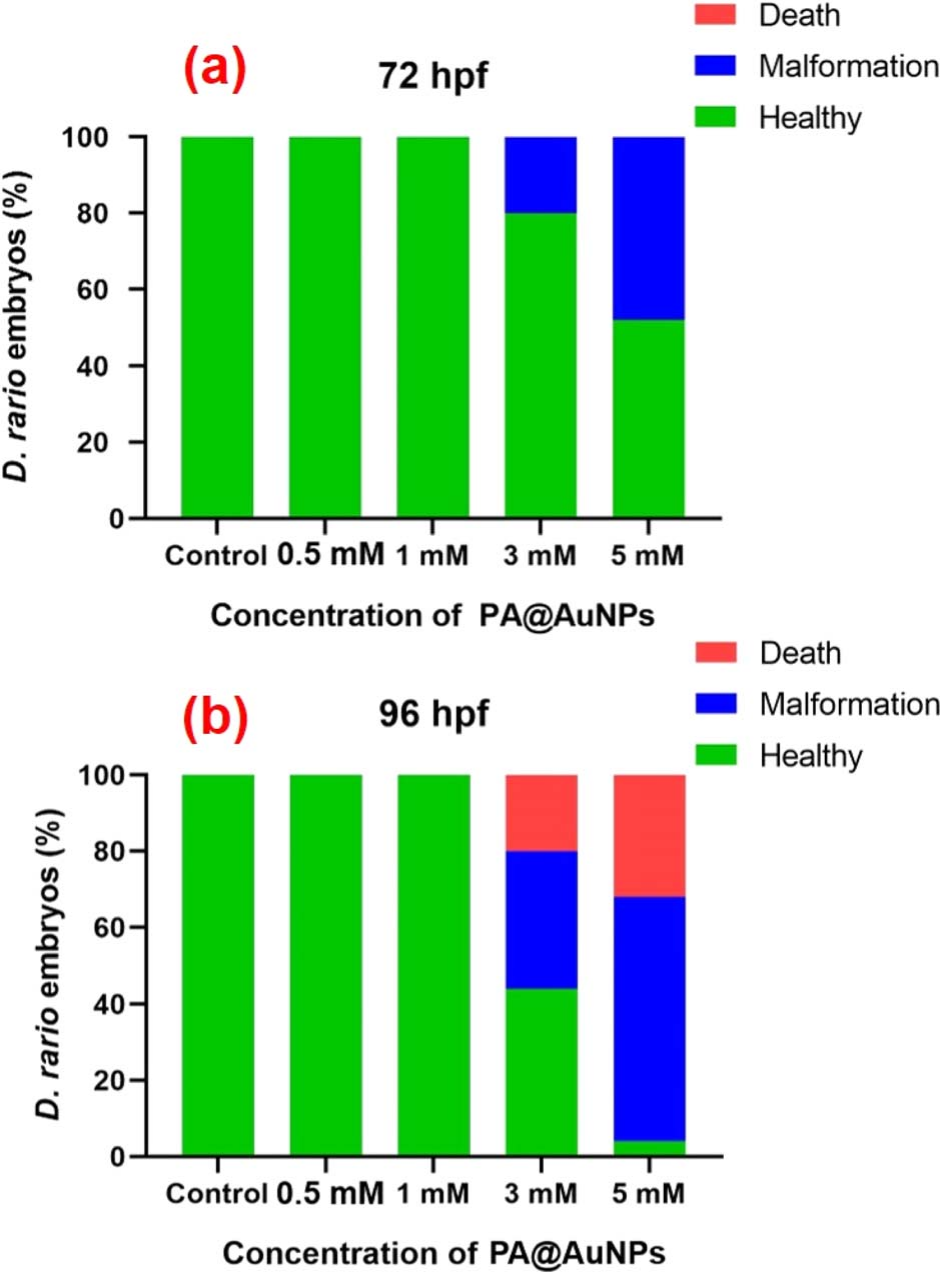
Morphological changes in zebrafish embryos affected by various concentrations of PA@AuNPs (0.5, 1, 3, and 5 mM). The morphological changes were observed at (a) 72 hpf, and (b) 96 hpf.

**Fig. 10:**
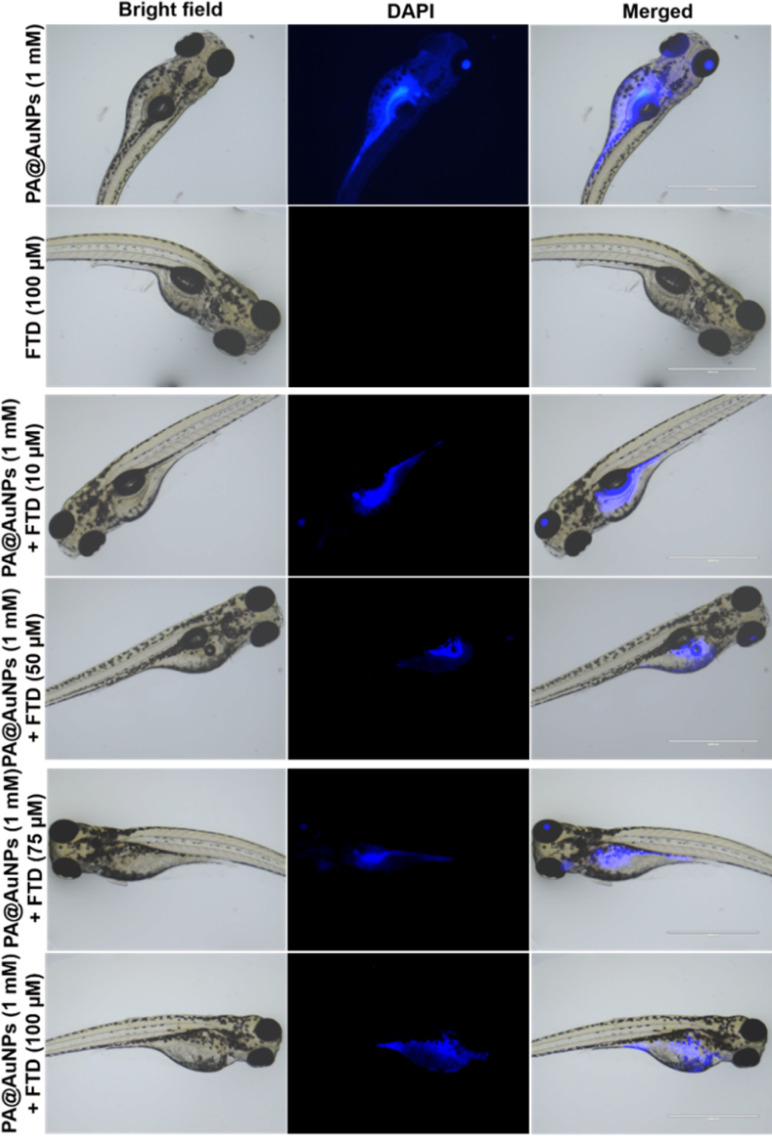
Brightfield and fluorescence images of zebrafish embryos after 24 h incubation with PA@AuNPs (1 mM) with increasing concentrations of FTD (10, 50, 75, and 100 μM).

The bioimaging study with zebrafish embryos was carried out to highlight the practical applicability of PA@AuNPs for detecting FTD in biological systems. Before doing bioimaging, the toxicity profile of PA@AuNPs was assessed using zebrafish. Zebrafish embryos (*n* = 25 per group) were exposed to varied concentrations of PA@AuNPs (0.5, 1, 3, and 5 mM in embryonic media) on a Petri dish for up to 96 hours after fertilization (hpf). Embryos not exposed to PA@AuNPs were used as controls. Using an inverted microscope, the toxicity profile (deformity and death) of zebrafish embryos was examined at 24, 48, 72, and 96 hours post fertilization. Zebrafish embryos are considered dead when they disintegrate or clot during development, or when their heartbeat stops. The following characteristics indicate deformation during embryogenesis: non-tail separation, lack of somite development, translucent eyes, pericardial edema (PE), yolk sac edema (YSE), hyperemia, and spinal curvature (SC). Based on the toxicological assessment, the non-toxic concentration of PA@AuNPs was employed to detect the FTD. The 96 hpf zebrafish embryos were exposed to PA@AuNPs for 3 hours in the embryonic medium, twice rinsed with the embryonic medium, and then sensing was performed with various FTD concentrations (10, 50, 75, and 100 μM). There is no deformation as a result of PA@AuNPs exposure, although embryos die at 24 and 48 hours post fertilization. Deformities such as PE, YSE, lack of swim bladder (LSB), and SC accumulate in zebrafish embryos between 72 and 96 hours post-fertilization (Fig. 8 and 9a and b). Notably, embryos died at 96 hours post fertilization when exposed to a high dose of 5 mM PA@AuNPs solution. As a result, at lower concentrations of PA@AuNPs, zebrafish embryos are unaffected. Thus, bioimaging tests were performed at a lower concentration of PA@AuNPs (1 mM), and no deformities or embryos were observed. There was no fluorescence signal obtained after 24 hours of incubation with just FTD (Fig. 10). When zebrafish embryos were exposed to the PA@AuNPs solution, however, they produced a vivid blue fluorescence signal. Interestingly, after 24 hours of incubation with a mixture of PA@AuNPs and FTD solution, modest blue fluorescence was noticed throughout the zebrafish body, despite increasing the concentration of FTD (10, 50, 75, and 100 μM) (Fig. 10). These biological study results show that PA@AuNPs have good biocompatibility and a high potential for biosensing applications in living species that are physiologically similar to humans.

### 3.7. Theoretical studies of PA@AuNPs, FTD, and PA@AuNPs·FTD complex

**Fig. 11:**
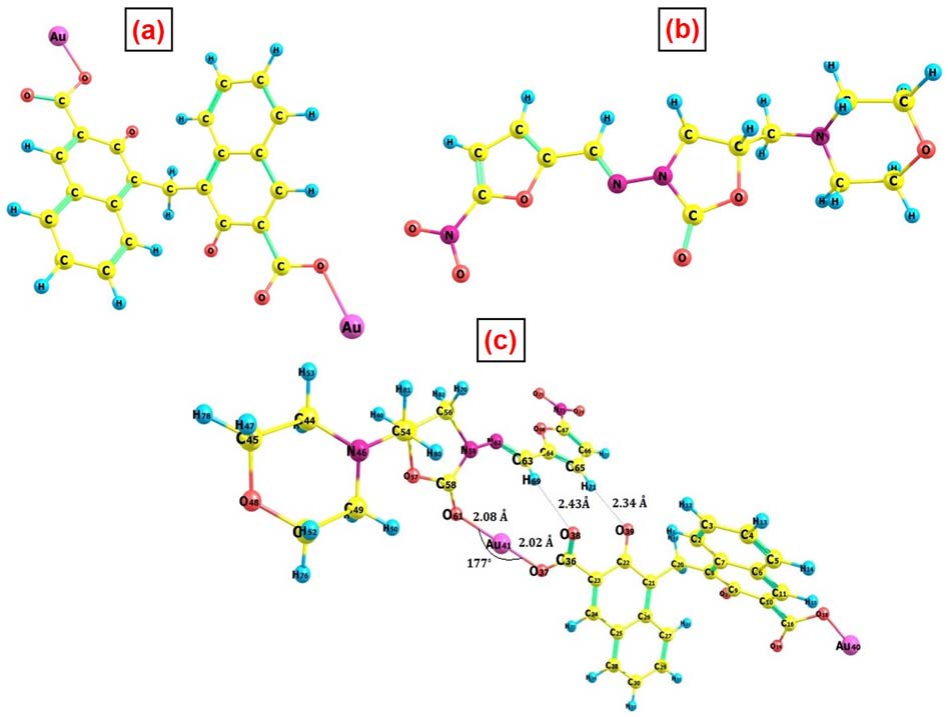
Optimized geometry of (a) PA@AuNPs, (b) FTD, and (c) PA@AuNPs·FTD complex at the B3LYP/6-311G(d,p) level of theory.

To corroborate all of the aforementioned experimental findings, the interaction between PA@AuNPs and FTD was studied using density functional theory at the B3LYP^49,50^/6-311G(d,p)^51^ level of theory. The ECP basis set, def2-TZVPP,^49^ was used for the Au atom. Fig. 11 depicts the optimal geometry of the isolated PA@AuNPs, FTD, and interacting complex, whereas Table SI2‡ lists the selected structural parameters. The optimized structure shows that the FTD interacts with PA@AuNPs through an O–Au covalent bond with a length of 2.08 Å. The bond length of the corresponding –C=O in the FTD molecule increases by 0.05 Å following the interaction. Additionally, two –O⋯H type hydrogen bond interactions were observed between the oxygen atoms of PA@AuNPs and the hydrogen atoms of the FTD, with distances of 2.43 and 2.33 Å. Fig. 10 shows that in both bare PA@AuNPs and the interaction complex, PA@AuNPs·FTD, naphthalene moieties with –COOAu functional groups were present in opposite directions. Binding another FTD at the –COOAu functional group on the other side of the PA@AuNPs verifies the formation of a ground state complex (PA@AuNPs·FTD), consistent with previous spectroscopic results.

The thermodynamic parameters of the interacting complex are generated in a strongly exothermic process with an enthalpy of −34.35 kcal mol^−1^ and an exoergic value of −20.86 kcal mol^−1^. The ground state density plot for the frontier molecular orbital, highest occupied molecular orbital (HOMO), lowest unoccupied molecular orbital (LUMO), and two singly occupied molecular orbitals (SOMO) were calculated using the optimized geometry of the complex PA@AuNPs·FTD (Fig. SI14‡), and the density plot is plotted with a contour of 0.03 a.u. SOMOs were found on naphthalene rings and the O-atom of PA@AuNPs. The HOMO is focused on the morpholine group of FTD, while the LUMO is localized on the free –COOAu functional group of PA@AuNPs in the interacting complex. The HOMO–LUMO energy gap for the interacting complex was found to be 2.47 eV. The vibrational frequency calculation done at the same theoretical level proved that the optimized geometries have a minimum energy point with all positive frequencies. Fig. SI13‡ depicts the theoretically estimated and simulated vibrational FTIR spectra of PA@AuNPs and FTD. In isolated FTD, a peak at 1901 cm^−1^ corresponds to the vibration of –C=O stretching, which is missing in the interacting complex, PA@AuNPs·FTD. The interaction of PA@AuNPs with FTD increases the strength of the peak at 1682 cm^−1^, which corresponds to the stretching vibration of –C–O. This confirms the conversion of a double bond into a single bond between atoms C58 and O61.

## 4. Conclusions

In summary, we are glad to provide the first report on PA@AuNPs as a novel fluorescent probe for ultra-sensitive detection of FTD (LoD 6.07 nM; *K*_a_ = 1.0595 × 10^2^ M^−1^) in blood serum. The quantum yield was lowered when increasing the concentration of FTD inside PA@AuNPs (*Φ*_F_ 3.36% → 0.35%). The UV-visible and fluorescence titrations show that both absorbance and fluorescence intensity decrease due to the establishment of a non-fluorescent complex, PA@AuNPs·FTD, which is verified by FTIR measurements. The decrease in emission intensity of PA@AuNPs with increasing FTD concentration was supported by a time-resolved fluorescence lifetime analysis (10.04 → 6.85 ns). Furthermore, the selectivity, and interference experiment results show that PA@AuNPs have great selectivity against other interfering compounds, and the time effect shows that PA@AuNPs can detect FTD within a minute. The cytotoxic reactivity results of PA@AuNPs against zebrafish embryos demonstrate that PA@AuNPs have biological applications. In addition, bioimaging of FTD in zebrafish demonstrates that PA@AuNPs could be employed as a sensor probe to detect and image FTD in humans under physiological conditions. To the best of our knowledge, this is the first and best report on fluorescent functionalized AuNPs as a probe for detecting FTD, an oral poultry medication, at nanomolar concentrations (Table SI3‡). The DFT calculation aids in the discovery of surface interactions between PA@AuNPs and FTD, as well as the possibility of synthesized PA@AuNPs to detect FTD in physiological systems. Based on the above findings, we confidently propose that PA@AuNPs could be an exciting material for poultry applications.

Even though a novel and straightforward technique for detecting FTD in blood serum has been proposed, the following challenges still need to be resolved: (i) an optimized synthesis method for the reliable and uniform size and shape of PA@AuNPs; (ii) simultaneous detection of multiple similar types of analytes; (iii) pH dependability; and (iv) require signal amplification techniques to increase sensitivity. Nevertheless, considerable systematic further interdisciplinary research can solve these aforementioned challenges.

## Data availability

All the results of this study are available within the article.

## Author contributions

A. Sowndarya: conceptualization, methodology, experiment, data curation, formal analysis, and writing-original draft. T. Daniel Thangadurai: conceptualization, writing-review & editing and supervision, validation, investigation, and data curation. N. Manjubaashini: formal analysis, and review the manuscript. M. Pavithrakumar and Senthilkumar: theoretical studies. D. Nataraj: TCSPC analysis and data validation. K. Kadirvelu and K. Naveen Kalagatur: bioimaging studies.

## Conflicts of interest

There is no conflict of interest to declare.

## Supplementary Material

RA-014-D4RA04293J-s001
