# Supplementary material for: Surface-designed AuNPs-based fluorescent probe for ultra-sensitive detection of oral poultry antibacterial drug furaltadone via intermolecular hydrogen bonding
Source: RSC Adv. 2024 Sep 4;14(38):28224–33. doi: 10.1039/d4ra04293j (PMC11372455; doi:10.1039/d4ra04293j)
Supplement: RA-014-D4RA04293J-s001 [file RA-014-D4RA04293J-s001.pdf]

Supplementary Information (SI) for RSC Advances.
This journal is © The Royal Society of Chemistry 2024

Supporting Information

Surface-designed AuNPs-based Fluorescent Probe for Ultra-sensitive detection of Oral Poultry Antibacterial drug Furaltadone via Intermolecular Hydrogen Bonding†

A. Sowndarya,[1] T. Daniel Thangadurai,[1*] N. Manjubaashini,[2] M. Pavithrakumar[3], K. Senthilkumar,[3] D. Nataraj,[3] K. Kadirvelu,[4] K. Naveen Kalagatur[4]

[1]Department of Chemistry, and Centre for Research and Development, KPR Institute of Engineering and Technology, Coimbatore 641407, India.

[2]National Centre for Nanoscience and Nanotechnology, University of Madras, Chennai 600025, India.

[3]Department of Physics, and [4]DRDO-Life Sciences, Bharathiar University, Coimbatore 641046, India.

*Corresponding author: Email: danielthangadurai.t@kpriet.ac.in.: Tel.: +91 422 263 5600.

1. Experimental Section

1.1. Preparation of Stock solution

A stock solution of PA@AuNPs and FTD was prepared by using double distilled water. The 1 mM working solution of PA@AuNPs was obtained by dilution of synthesized PA@AuNPs with 50 mL H_2O. Simultaneously, the 100 μM of FTD stock solution (0.0032 g in 100 mL H_2O) was prepared in a 50 mL beaker.

2. Characterization

2.1. Fluorescence studies

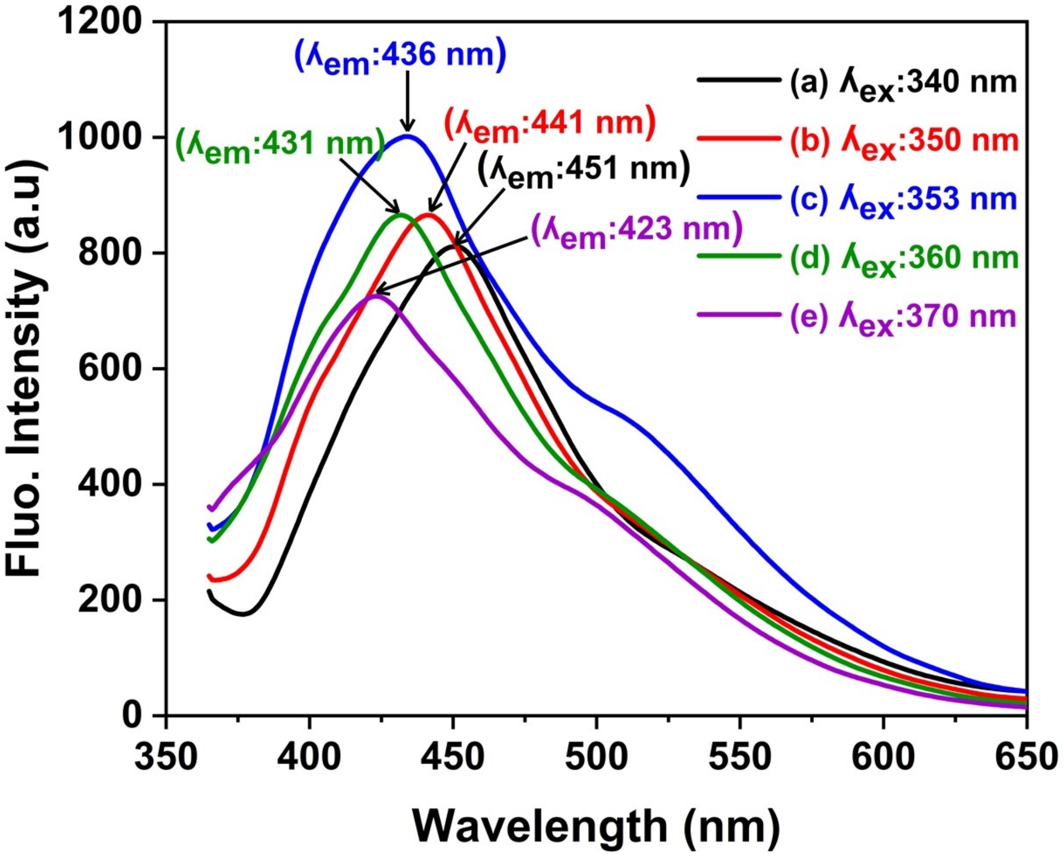

Figure SI1. Fluorescence emission spectrum of PA@AuNPs using different excitation (λ_{ex} 340, 350, 353, 360, and 370 nm).

2.2. Selectivity study

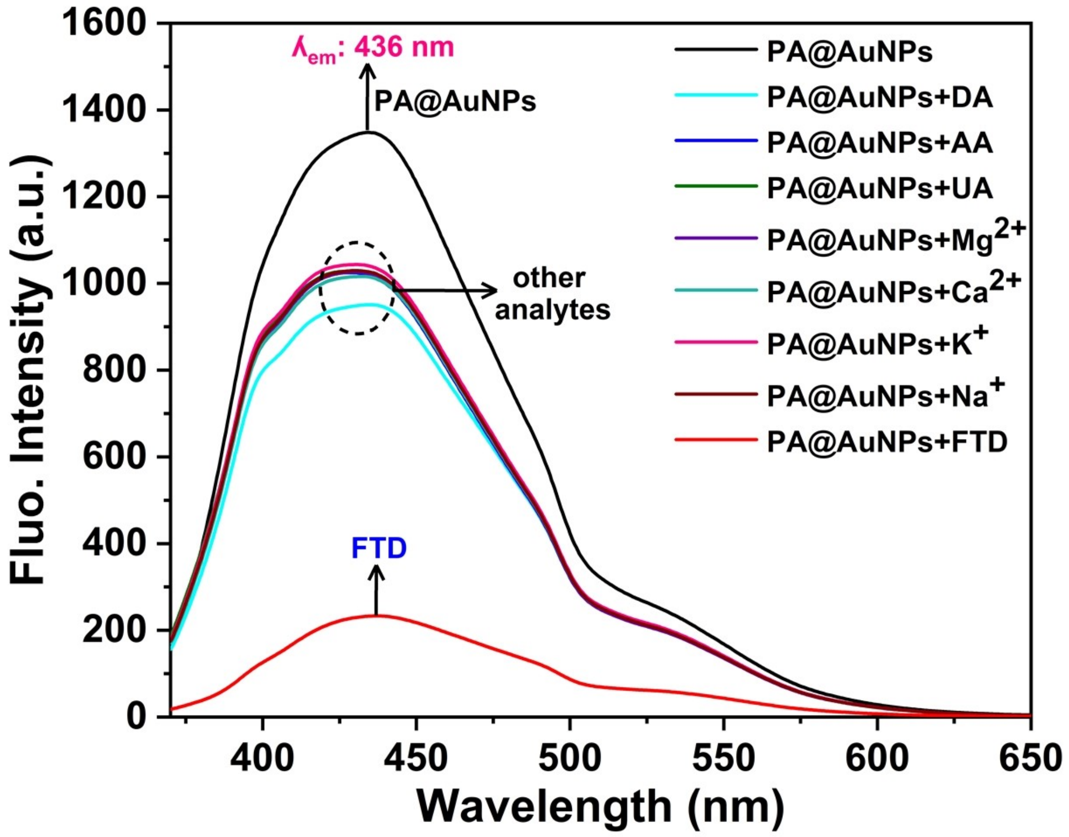

Figure SI2. Fluorescence emission intensity changes of PA@AuNPs [1.0 mM] upon addition of various analytes [100 μM] (DA – Dopamine, AA – Ascorbic acid, UA – Uric acid, Mg^{2+} - Magnesium ions, Ca^{2+} - Calcium ions, K^{+} - Potassium ions, Na^{+} - Sodium ions, and FTD – Furaltadone) (λ_{ex} 353 nm; slit width 5 nm).

2.3. Binding constant and LoD values

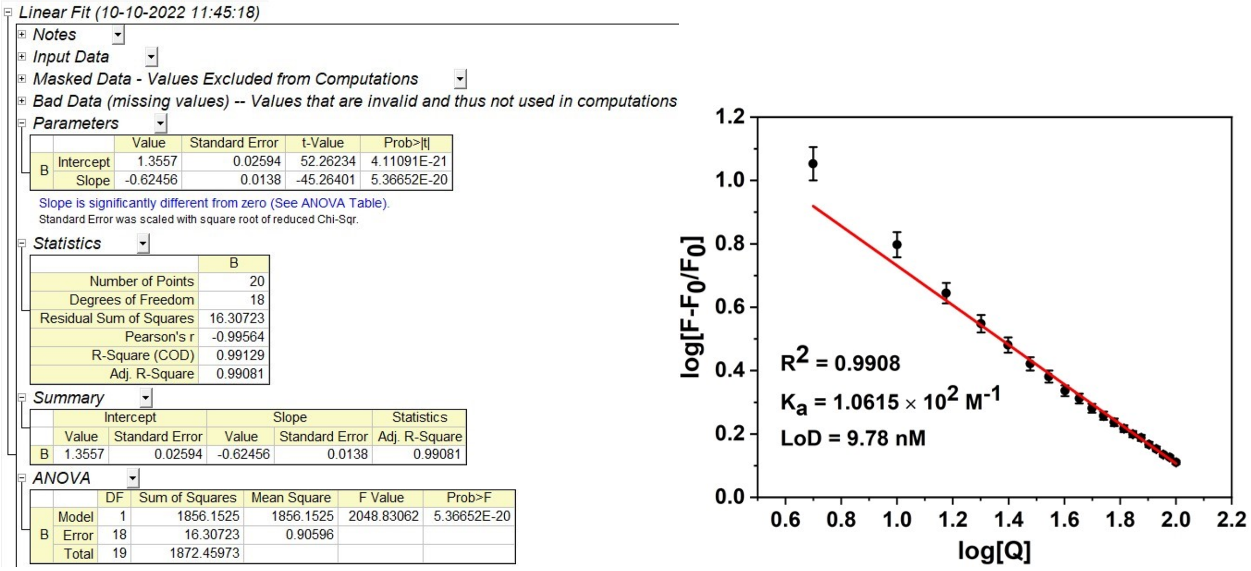

Linear Fit (10-10-2022 11:45:18)

Notes

Input Data

Masked Data - Values Excluded from Computations

Bad Data (missing values) -- Values that are invalid and thus not used in computations

Parameters

		Value	Standard Error	t-Value	Prob>|t|
B	Intercept	1.3557	0.02594	52.26234	4.11091E-21
	Slope	-0.62456	0.0138	-45.26401	5.36652E-20

Slope is significantly different from zero (See ANOVA Table).
Standard Error was scaled with square root of reduced Chi-Sqr.

Statistics

	B
Number of Points	20
Degrees of Freedom	18
Residual Sum of Squares	16.30723
Pearson's r	-0.99564
R-Square (COD)	0.99129
Adj. R-Square	0.99081

Summary

	Intercept		Slope		Statistics
	Value	Standard Error	Value	Standard Error	Adj. R-Square
B	1.3557	0.02594	-0.62456	0.0138	0.99081

ANOVA

		DF	Sum of Squares	Mean Square	F Value	Prob>F
B	Model	1	1856.1525	1856.1525	2048.83062	5.36652E-20
	Error	18	16.30723	0.90596		
	Total	19	1872.45973			

Figure SI3. Fluorescence emission intensity changes of PA@AuNPs upon the addition of FTD log[F-F_0/F_0] against log[Q], binding constant (K_a) calculated through linear fit (R^2 = 0.9908; STD = 3%).

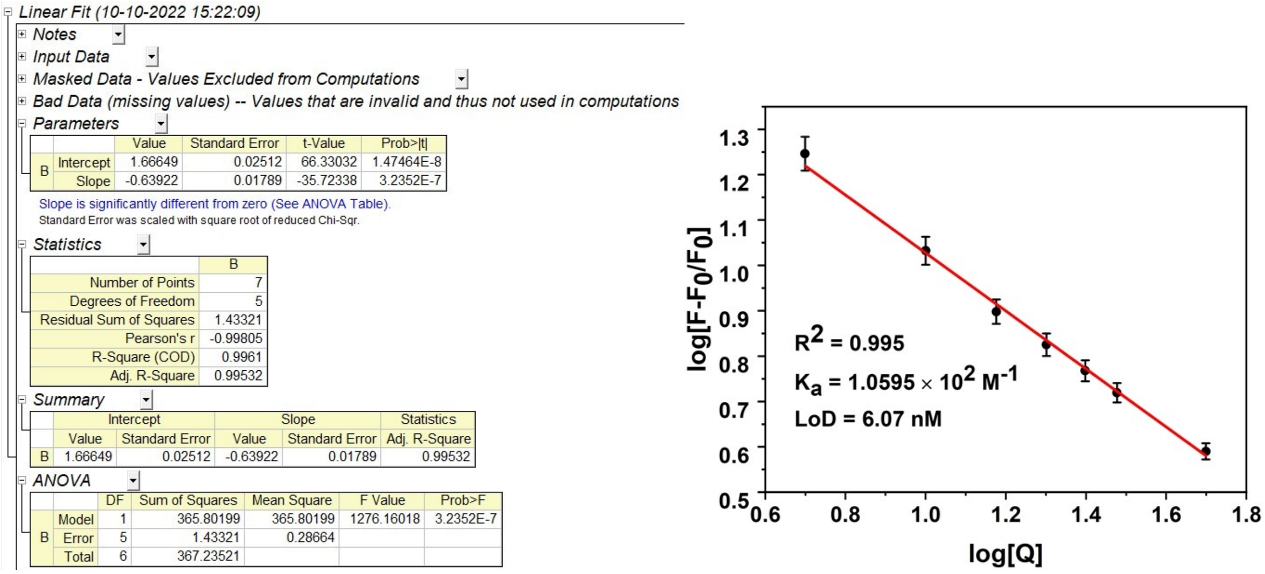

Linear Fit (10-10-2022 15:22:09)

Notes

Input Data

Masked Data - Values Excluded from Computations

Bad Data (missing values) -- Values that are invalid and thus not used in computations

Parameters

		Value	Standard Error	t-Value	Prob>|t|
B	Intercept	1.66649	0.02512	66.33032	1.47464E-8
	Slope	-0.63922	0.01789	-35.72338	3.2352E-7

Slope is significantly different from zero (See ANOVA Table).
Standard Error was scaled with square root of reduced Chi-Sqr.

Statistics

	B
Number of Points	7
Degrees of Freedom	5
Residual Sum of Squares	1.43321
Pearson's r	-0.99805
R-Square (COD)	0.9961
Adj. R-Square	0.99532

Summary

	Intercept		Slope		Statistics
	Value	Standard Error	Value	Standard Error	Adj. R-Square
B	1.66649	0.02512	-0.63922	0.01789	0.99532

ANOVA

		DF	Sum of Squares	Mean Square	F Value	Prob>F
B	Model	1	365.80199	365.80199	1276.16018	3.2352E-7
	Error	5	1.43321	0.28664		
	Total	6	367.23521			

Figure SI4. Fluorescence emission intensity changes of PA@AuNPs upon the addition of blood serum and [FTD] log[F-F_0/F_0] against log[Q], binding constant (K_a) calculated through linear fit (R^2 = 0.995; STD = 3%).

2.4. Interference study

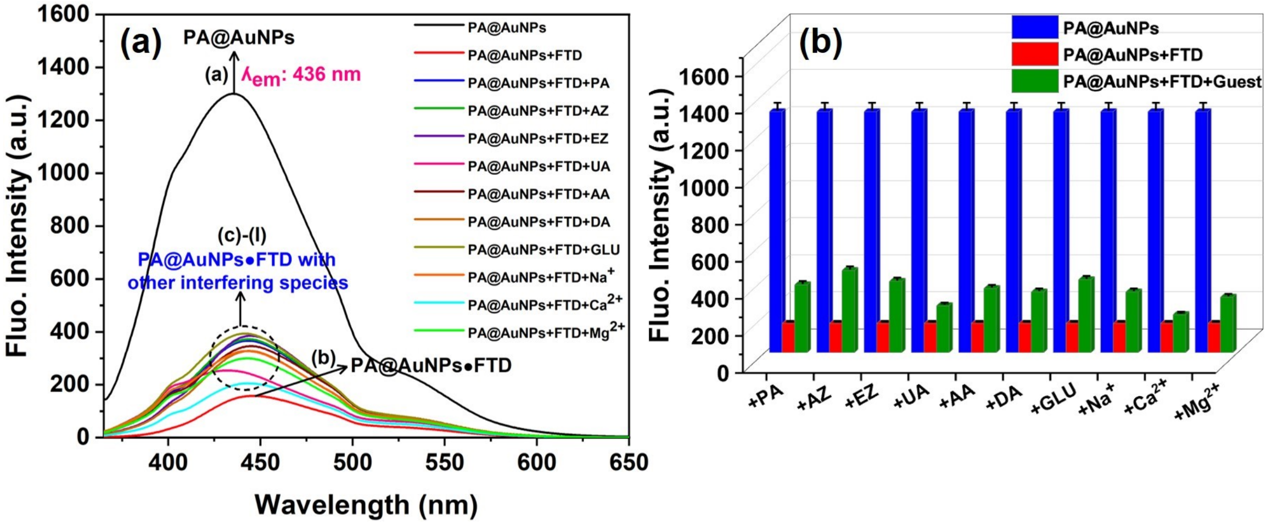

Figure SI5. (a) Fluorescence response for PA@AuNPs [1.0 mM] with other interfering compounds (100 μM) in the presence of FTD (1 μM)-(b), paracetamol (PR)-(c), azithromycin (Az)-(d), erythromycin (EZ)-(e), uric acid (UA)-(f), ascarbic acid (AA)-(g), dopamine (DA)-(h), glucose (GLU)-(i), sodium ions (Na^{+})-(j), calcium ions (Ca^{2+})-(k), magnesium ions (Mg^{2+})-(l), (b) Corresponding bar diagram (STD = 3%).

2.5. Time effect

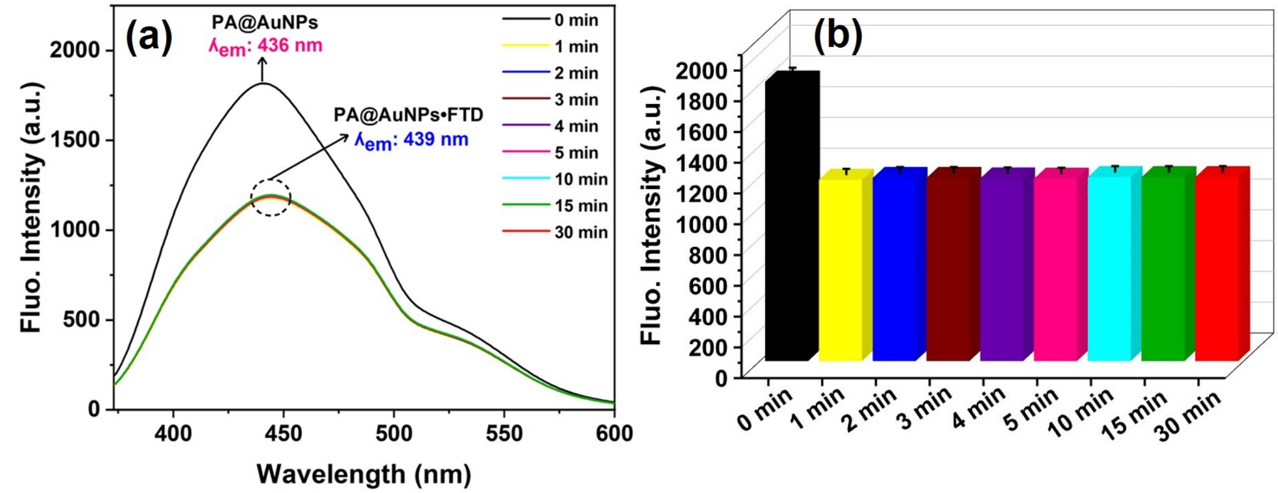

Figure SI6. (a) Time effect on the fluorescence intensity of PA@AuNPs [1.0 mM] upon sensing of FTD (100 μM) (λ_{ex} 353 nm; slit width 5 nm). (b) Corresponding bar diagram (STD = 3%).

2.6. Reversibility study

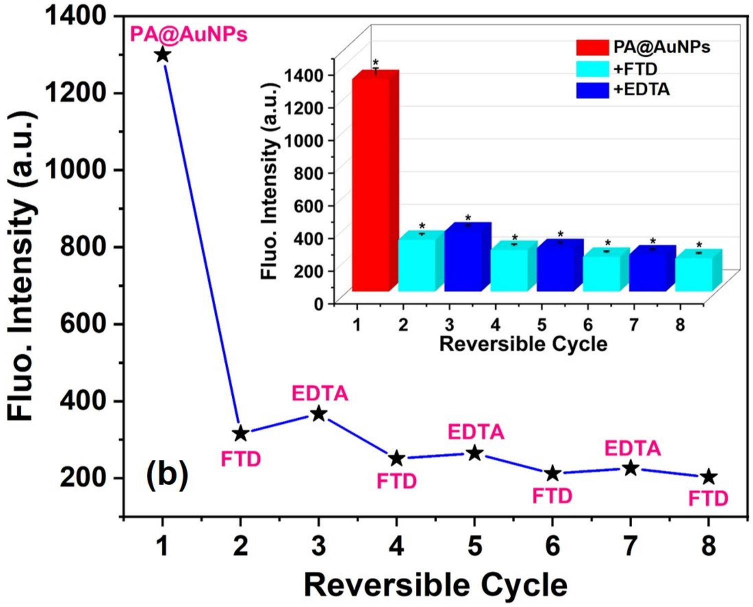

Figure SI7. (a) Reversible fluorescence emission spectrum of PA@AuNPs (1.0 mM) with the alternative addition of 100 μM FTD and EDTA (λ_{ex} 353 nm; slit width for both excitation and emission is 5 nm). (b) Corresponding reversible cycle, (inset (b) the corresponding bar diagram) (STD = 3%).

2.7. pH effect

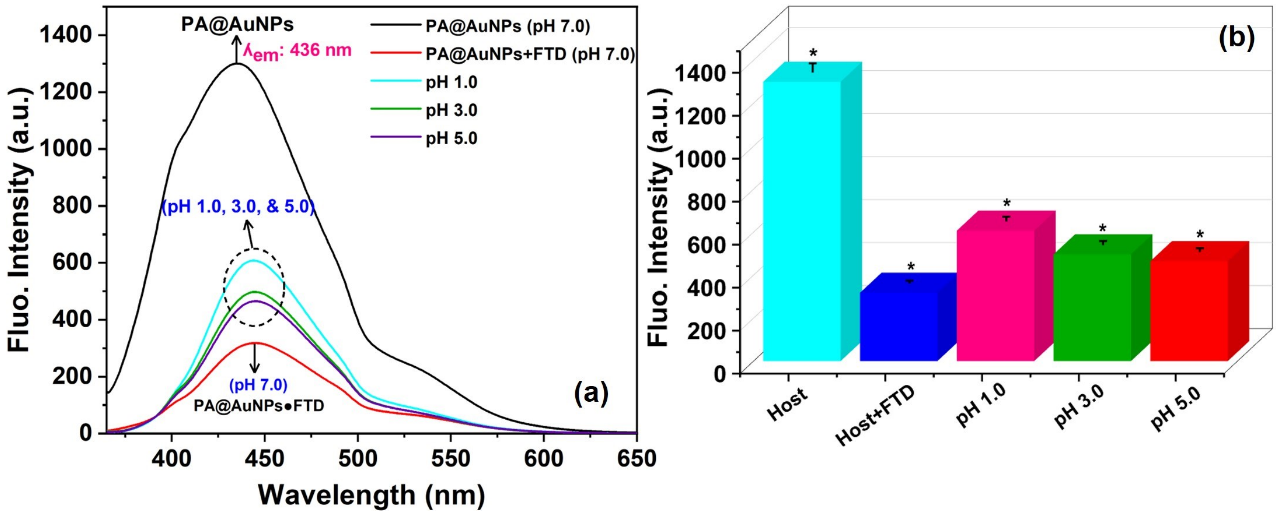

Figure SI8. (a) Fluorescence emission intensity changes of PA@AuNPs•FTD in acidic pH condition (pH 1 to 5) (λ_{ex} 353 nm; slit width for both excitation and emission is 5 nm), and (b) Corresponding bar diagram (STD = 3%).

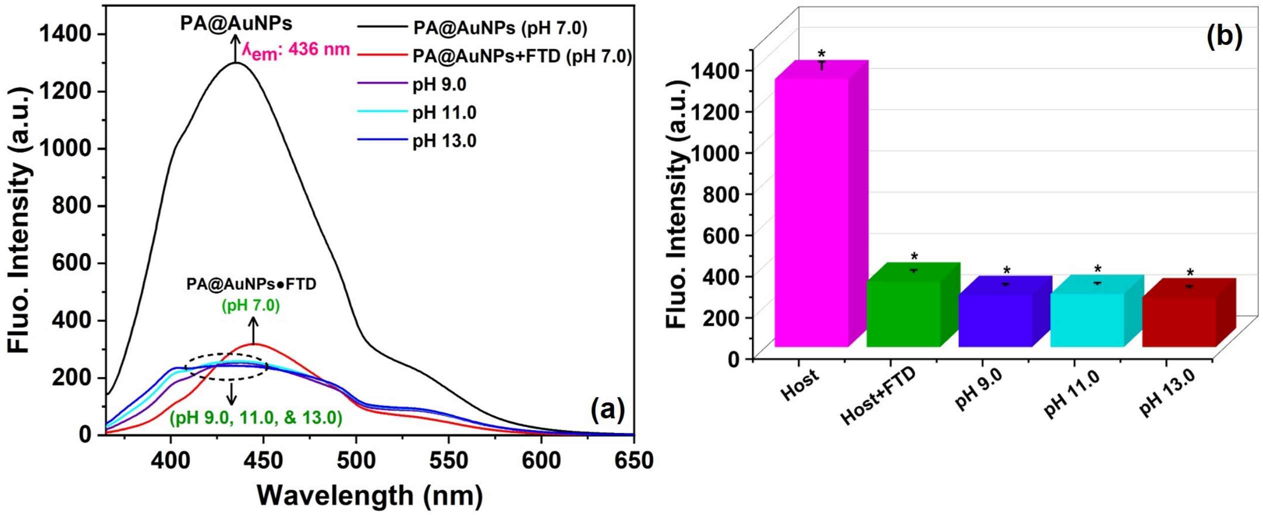

Figure SI9. (a) Fluorescence emission intensity changes of PA@AuNPs•FTD in basic pH condition (pH 9 to 13) (λ_{ex} 353 nm; slit width for both excitation and emission is 5 nm), and (b) Corresponding bar diagram (STD = 3%).

2.8. HRTEM analysis

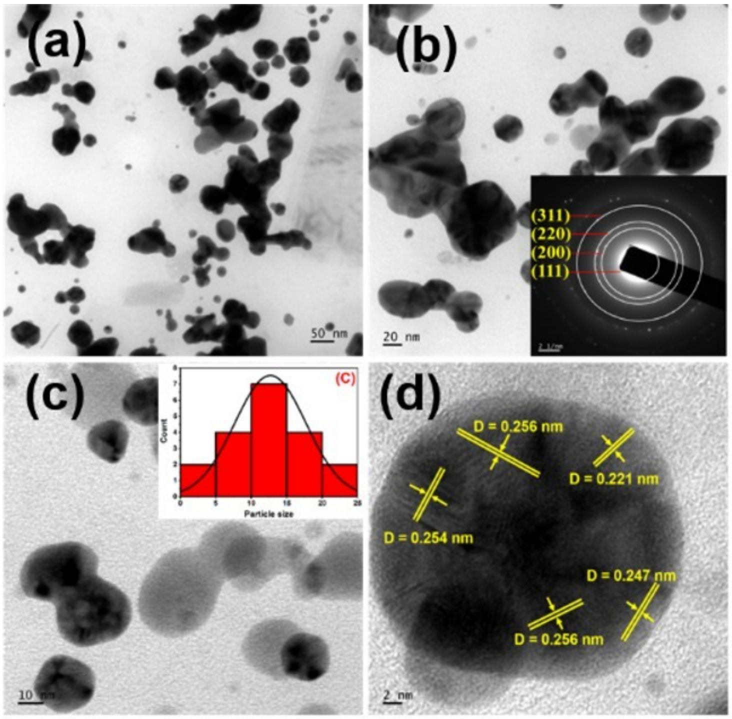

Figure SI10. (A) HRTEM images of PA@AuNPs in a different scale (a→d; 50, 20, 10, and 2 nm), (inset b) SAED pattern of PA@AuNPs, (inset c) histogram of PA@AuNPs of the average diameter of 10-15 nm, and (d) fringes with average 'd' space value of 0.248 nm;

2.9. FESEM and AFM analysis

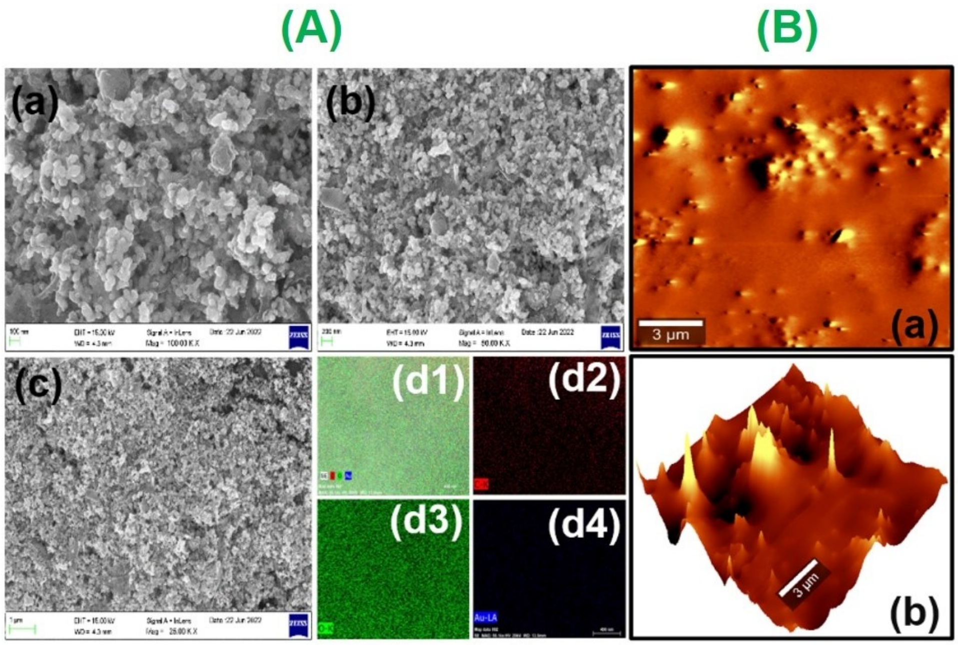

Figure SI11. (A) FESEM images of PA@AuNPs (a) 1 µm, (b) 200 nm, and (c) 100 nm scale, respectively, (d) elemental mapping of PA@AuNPs (d1), C (d2), O (d3), Au (d4); and (B) AFM images of PA@AuNPs in (a) 2D, and (b) 3D modes.

2.10. EDAX analysis

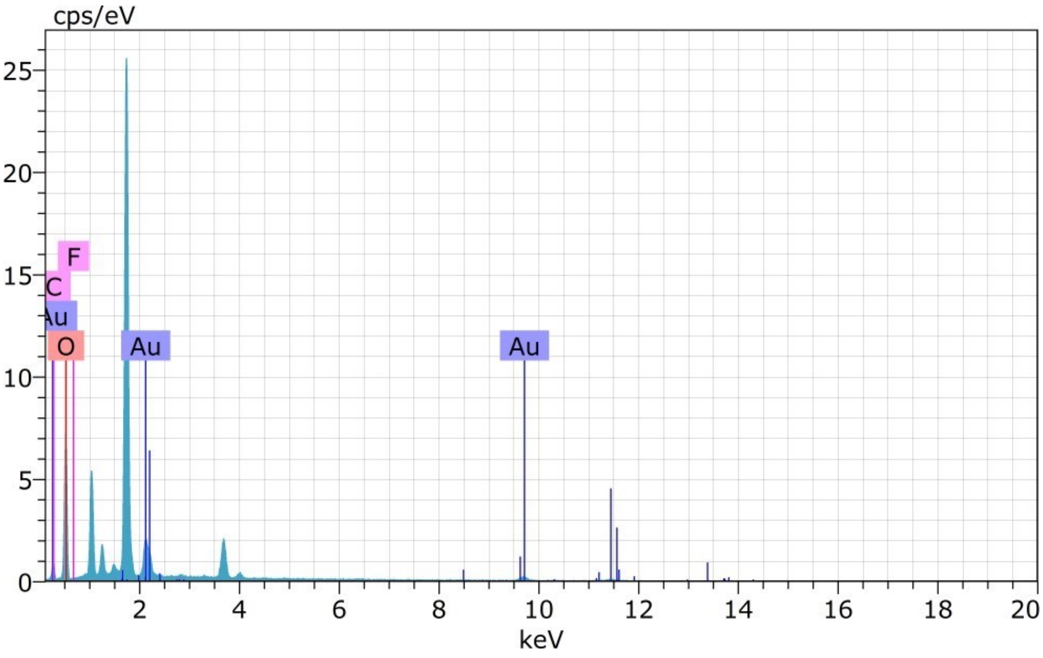

Figure SI12. EDAX analysis of PA@AuNPs•FTD.

Table SI1. Weight percentage of the elements present in PA@AuNPs•FTD.

Spectrum: BM 3084

El	AN	Series	unn. C [wt.%]	norm. C [wt.%]	Atom. C [at.%]	Error (1 Sigma) [wt.%]
O	8	K-series	30.12	60.55	78.50	3.92
Au	79	L-series	14.16	28.47	3.00	0.55
C	6	K-series	5.11	10.27	17.73	1.01
F	9	K-series	0.35	0.71	0.78	0.19
		Total:	49.74	100.00	100.00	

2.11. UV-Vis spectral studies

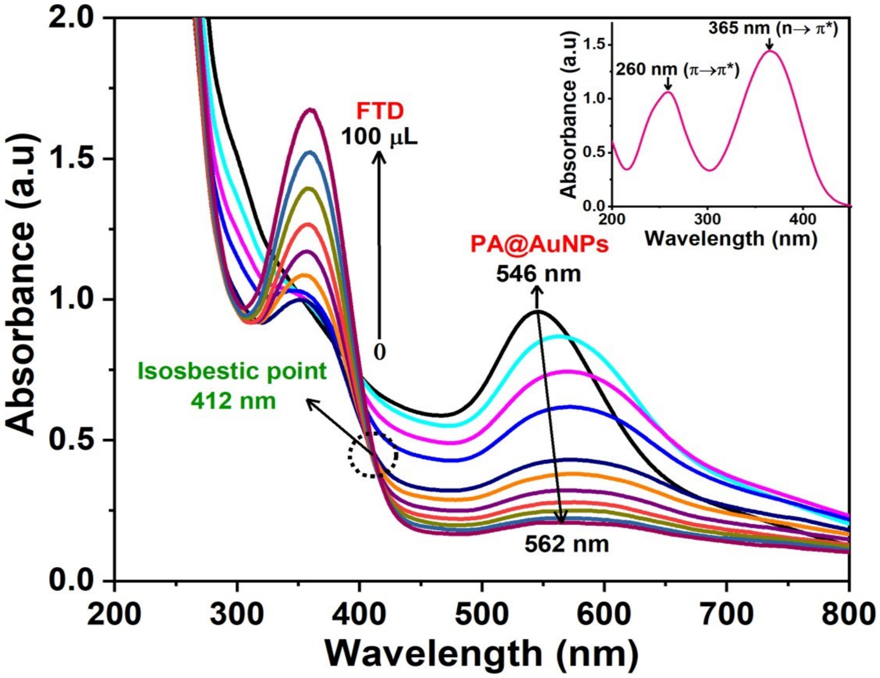

Figure SI13. UV-visible absorption spectrum for the addition of FTD (1.0 mM) (0 – 100 μL) into the PA@AuNPs solution (1.0 mM) (inset: UV-visible absorption spectrum of FTD).

Linear Fit (20-10-2022 09:46:32)

Notes

Input Data

Masked Data - Values Excluded from Computations

Bad Data (missing values) -- Values that are invalid and thus not used in computatio

Parameters

		Value	Standard Error	t-Value	Prob>|t|
B	Intercept	0.07553	0.05753	1.31285	0.24626
	Slope	0.01743	9.30716E-4	18.72292	8.003E-6

Slope is significantly different from zero (See ANOVA Table).

Standard Error was scaled with square root of reduced Chi-Sqr.

Statistics

	B
Number of Points	7
Degrees of Freedom	5
Residual Sum of Squares	4.73095
Pearson's r	0.99294
R-Square (COD)	0.98594
Adj. R-Square	0.98312

Summary

	Intercept		Slope		Statistics
	Value	Standard Error	Value	Standard Error	Adj. R-Square
B	0.07553	0.05753	0.01743	9.30716E-4	0.98312

ANOVA

		DF	Sum of Squares	Mean Square	F Value	Prob>F
B	Model	1	331.6845	331.6845	350.54769	8.003E-6
	Error	5	4.73095	0.94619		
	Total	6	336.41545			

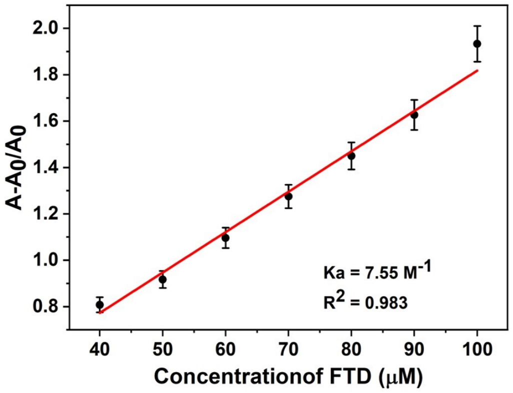

Figure SI14. Absorption changes of PA@AuNPs (1 mM) [A-A_0/A_0] upon the addition of FTD (1 mM) FTD [Q], binding constant (K_a) calculated through linear fit (R^2 = 0.983) (STD = 3%).

2.12. Zeta Potential

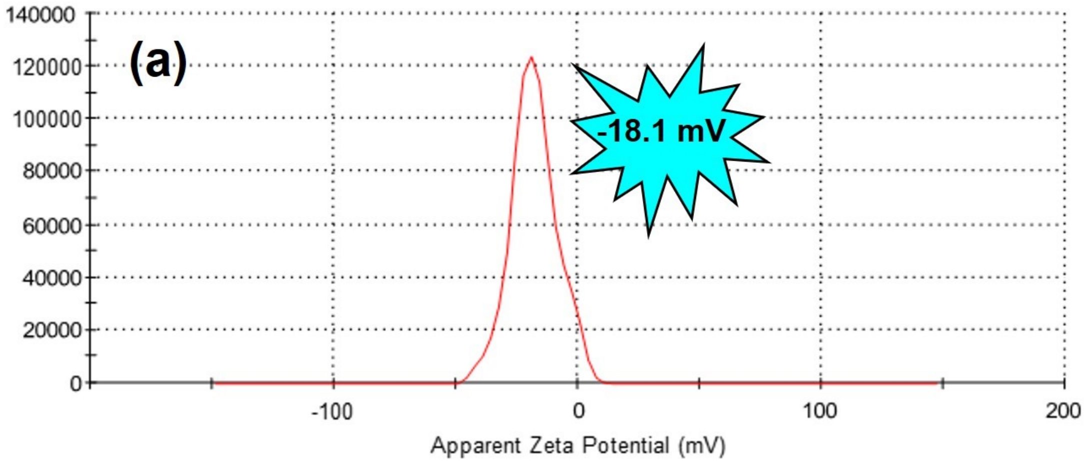

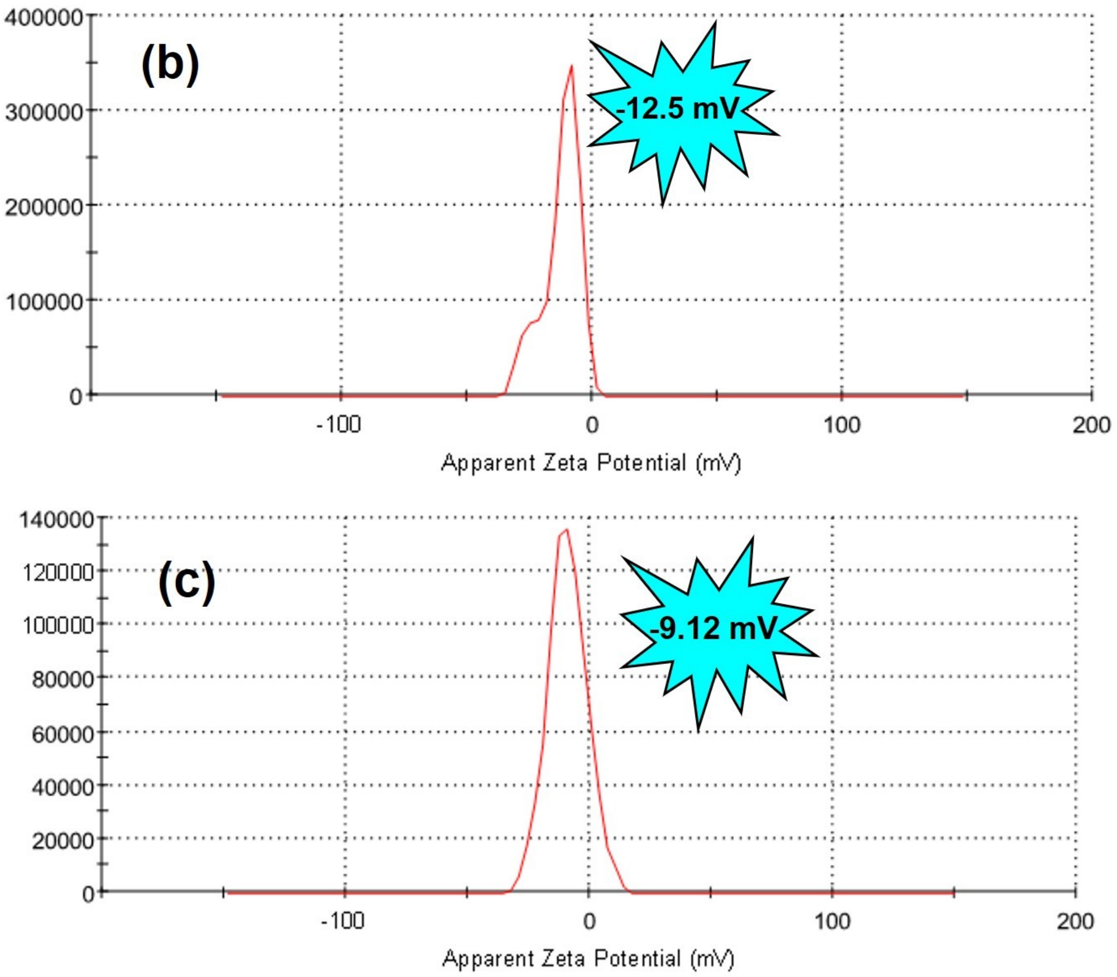

Figure SI15. (a) Zeta potential of bare PA@AuNPs, (b) PA@AuNPs+FTD (0.5 μM), and (c) PA@AuNPs+FTD (0.75 μM) .

3. DFT Calculation for PA@AuNPs•FTD

The interaction between PA@AuNPs and FTD molecules was investigated by using density functional theory, B3LYP/6-311G(d,p) level of theory. The ECP basis set, def2-TZVPP was employed for the Au atom. The selected structural parameters are listed in Table SI2.

Table SI2. The selected geometrical parameters (bond length in Å, angle in degrees) of isolated PA@AuNPs, FTD molecules, and interacting complex

Parameter	Isolated molecules		Interacting complex
	FTD	PA@AuNPs	
**R1(C58-O61)**	**1.18**	**-**	**1.23**
R2(C63-H69)	1.09	-	1.09
R3(C65-H71)	1.08	-	1.08
R4(O61-Au41)	-	-	2.08
**R5(O37-Au41)**	**-**	**2.05**	**2.02**
R6(C36-C37)	-	1.31	1.31
R7(C36-C38)	-	1.22	1.23
R8(C22-O39)	-	1.24	1.25
R9(C9-O17)	-	1.24	1.24
R10(C16-O19)	-	1.21	1.21
**ϴ1(O61-Au41-O37)**	**-**	**-**	**177**
**ϴ2(C36-O38…H69)**	**-**	**-**	**127**
**ϴ3(C22-O39…H71)**	**-**	**-**	**130**
ϴ4(C58-O61-Au41)	-	-	131.1

ϴ5(Au41-O37-C36)	-	110.7	115
ϴ6(O37-C36-C23)	-	114	112.9
ϴ7(O38-C36-C23)	-	120.6	121.2
ϴ8(O39-C22-C23)	-	122.4	122
ϴ9(O61-C58-O57)	124.9	-	119.1
ϴ10(O61-C58-N59)	128	-	130.1
ϴ11(N62-C63-H69)	124	-	124.9
ϴ12(C64-C65-H71)	126.1	-	126.4
ϴ13(C8-C20-C21)	-	116.4	116.4
ϴ14(N57-N62-C63)	119.2	-	115.4
ϴ1(C58-O61-Au51-O37)	-	-	66.2
ϴ2(O61-Au41-O37-C36)	-	-	64
ϴ3(C22-C21-C20-C8)	-	112.7	113.5
ϴ4(C26-C21-C20-C8)	-	-71.2	-70.74
ϴ5(C7-C8-C20-C21)	-	-72.1	-70.6
ϴ6(C9-C8-C20-C21)	-	112.2	113.3

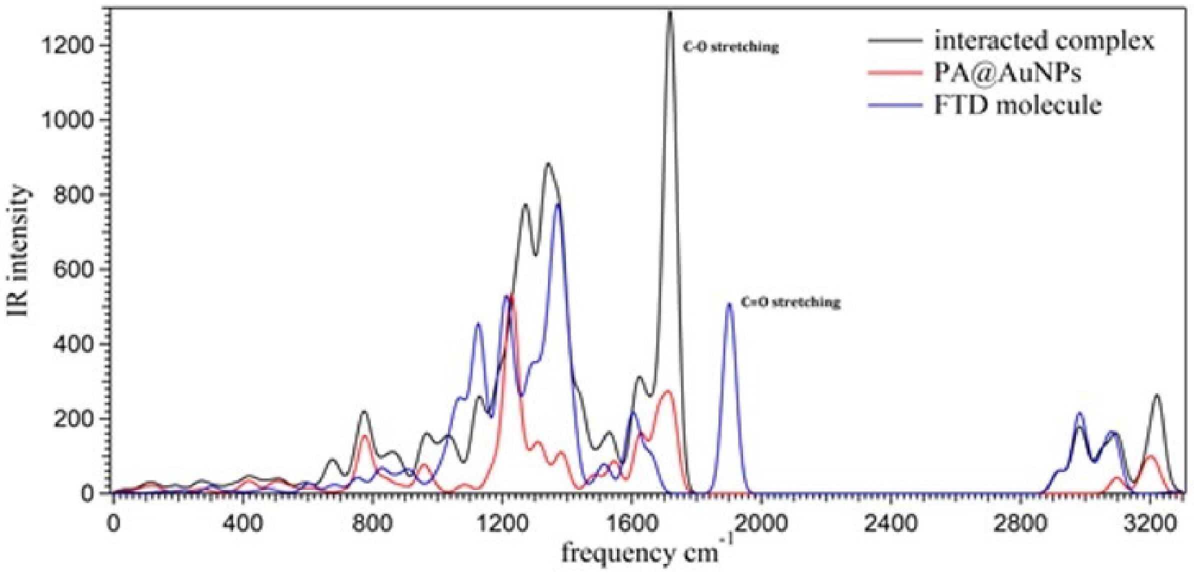

Figure SI16. The calculated FTIR spectrums for PA@AuNPs, Furaltadone (FTD), and product complex.

The ground state density plot for frontier molecular orbital, highest occupied molecular orbital (HOMO), lowest unoccupied molecular orbital (LUMO), and two singly occupied molecular orbitals (SOMO) were calculated based on the optimized geometry of the product complex. The plot was made with the contour value of 0.03 a.u. The SOMOs were localized on naphthalene rings and O^- atom of PA@AuNPs. Further, the HOMO is localized on the morpholine group of FTD and LUMO is localized on the free -COOAu functional group of PA@AuNPs in the interacting complex. The energy gap between HOMO and LUMO is found as 2.47 eV.

Figure SI17. The ground state density plot of SOMO, HOMO, and LUMO molecular orbitals of the studied interacting complex.

S. No.	Orbitals	Interacting complex (PA@AuNPs•FTD)
**1.**	**SOMO 1**	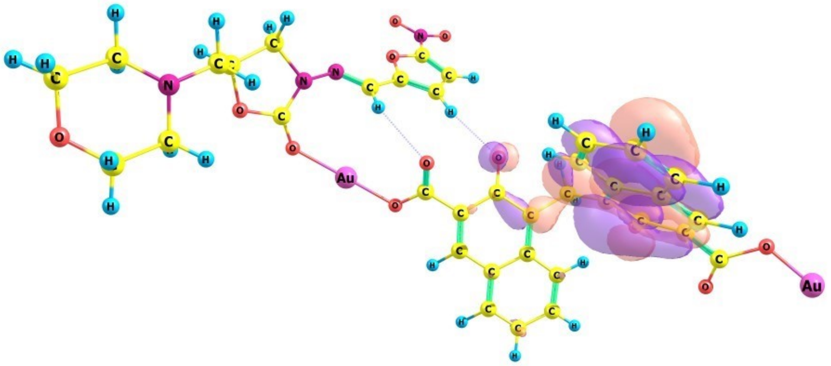
**2.**	**SOMO 2**	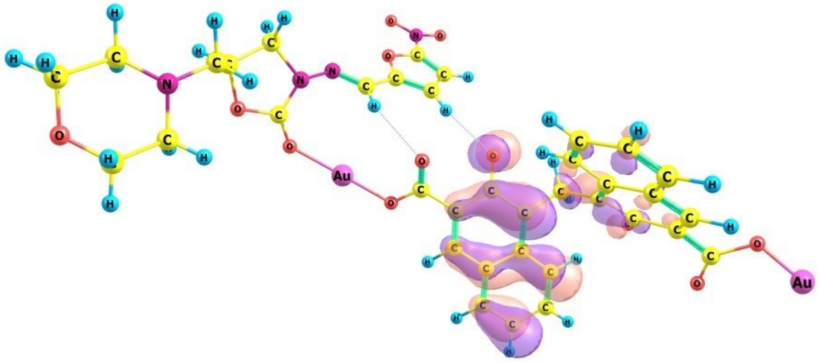
**3.**	**HOMO**	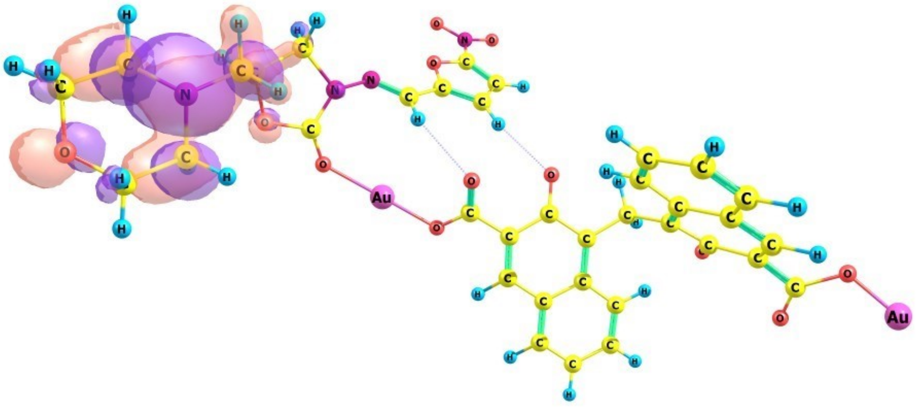

4.	LUMO	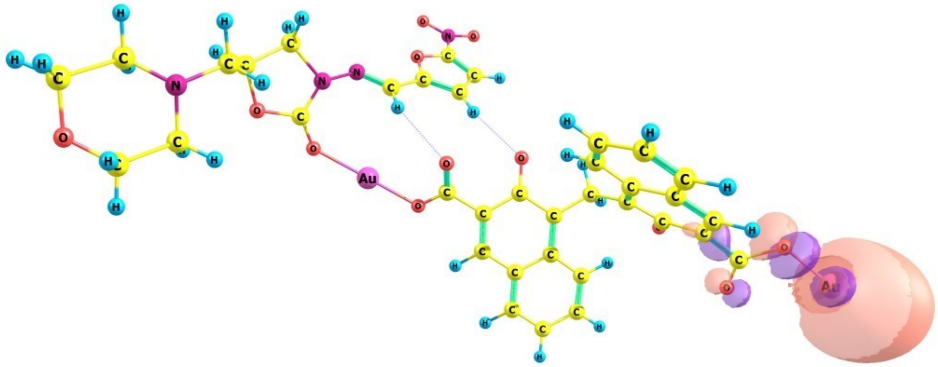

Table SI3. Comparison of nanostructured-based probes for furaltadone detection reported so far.

S. No.	Material Used & Publication details	LoD	Analyte Used	Method of detection	Reference No. (in SI)
1.	Gold nanosubstrates<br><br>*Eur Food Res Technol, 235 (2012) 555–561*	5 ppm	FTD & Furadantin	SERS	1
2.	M-MWCNT (Magnetic multi-wall carbon nanotubes)<br><br>*Water Science & Technology 70.6 (2014) 964-971*	320 $\mu g.L^{-1}$	FTD	Thermodynamic analysis	2
3.	MWCNT<br><br>(Multi-wall carbon nanotubes)<br><br>*Analytical and Bioanalytical Chemistry 410 (2018) 6573−6583*	0.012 µM	Nitrofuran drugs (NFT, FTD, FZD & NFZ)	Electrochemical sensor (DPV)	3
4.	Gold nanoparticles (AuNPs)<br><br>*Microchemical*	0.01 ng/mL	FTD	Signal enhanced ELISA	4

	Journal 159 (2020) 105414				
5.	FeVO/P-rGO NCs<br><br>*Applied Surface Science 569 (2021) 151046*	138 nM	FTD	Electrochemical (CV, DPV & amperometric studies)	5
6.	$SrMnO_3$/*f*-BN composite<br><br>*Catalysts 12 (2022) 1494*	2.0 nM	FTD	Electrochemical<br><br>(voltammetric detection)	6
7.	$CuCoO_2$<br><br>*Int. J. Electrochem. Sci., 17 (2022) 220644*	1.79 nM	FTD	Electrochemical (CV & DPV)	7
8.	Cu/Ni/TiO_2/MWCNTs nanocomposites<br><br>*Scientific Reports 12 (2022) 886*	0.0949 µM	FTD	Electrochemical & photocatalytic	8
9.	ZnO & $ZnCo_2O_4$<br><br>*Microchemical Journal 169 (2021) 106566*	1.46 nM & 34.1 nM	FTD	Electrochemical	9
10.	Co_2SnO_4/SnO_2 (Cobalt tin oxide/tin oxide)<br><br>*Journal of Alloys and Compounds 882 (2021) 160750*	39 nM	FTD	Electrochemical detection	10
11.	2-hydroxy-1-naphthaldehyde *Food Additives & Contaminants: Part A, 30 (2013) 2114−2122*	0.64 µg kg^{-1} & 0.15 µg kg^{-1}	FTD	HPLC and LC-MS/MS	11

12.	Histidine-Cu NCs<br>*Chemical Papers 76 (2022) 7855–7863*	0.9948 µM	FTD	Fluorescence spectroscopy	12
13.	Metal-regulated d^{10} coordination polymers constructed from bis(pyridyl)-bis(amide) ligands *New Journal of Chemistry 47 (2023) 9701-9707*	9.41×10^{-8} M	FTD	Fluorescence spectroscopy	13
14.	Tryptophan-protected gold nanoclusters *Spectrochimica Acta Part A: Molecular and Biomolecular Spectroscopy 308 (2024) 123748*	0.087 µM	FTD	Fluorescence spectroscopy	14
15.	PA@AuNPs	9.78 nM &<br>6.07 nM	FTD in Normal & Blood serum	Fluorescence spectroscopy	Current work

References

(1) Xie, Y.; Zhu, X.; Sun, Y.; Wang, H.; Qian, H.; Yao, W. Rapid detection method for nitrofuran antibiotic residues by surface-enhanced Raman Spectroscopy. *European Food Research and Technology* **2012**, 235, 555−561.

(2) Chen, X.; Xiong, Z. H. Kinetics and thermodynamics of the sorption of furaltadone from aqueous solutions on magnetic multi-walled carbon nanotubes. *Water science and technology* **2014**, 70, 964−971.

(3) Chiu, S-H.; Su, Y-L.; Le, A. V. T.; Cheng, S-H. Nanocarbon material-supported conducting poly (melamine) nanoparticle-modified screen-printed carbon electrodes for highly sensitive determination of nitrofuran drugs by adsorptive stripping voltammetry. *Analytical and bioanalytical chemistry* **2018**, 410, 6573−6583.

(4) Yan, C.; Teng, J.; Liu, F.; Yao, B.; Xu, Z.; Yao, L.; Chen, W. Signal amplified enzyme-linked immunosorbent assay with gold nanoparticles for sensitive detection of trace furaltadone metabolite. *Microchemical Journal* **2020**, 159, 105414−105419.

(5) Rajakumaran, R.; Babulal, S. M.; Chen, S. M.; Sukanya, R.; Karthik, R.; Shafi, P. M.; Shim, J. J.; Yo-Shiuan, C. Ingenious design of iron vanadate engulfed 3D porous reduced graphene oxide nanocomposites as a reliable electrocatalyst for the selective amperometric determination of furaltadone in aquatic environments. *Applied Surface Science* **2021**, 569, 151046−151058.

(6) Venkatesh, K.; Rajakumaran, R.; Chen, S. M.; Karuppasamy, P.; Banach, A.; Al-Onazi, W. A.; Sonadevi, S.; Krishnan, N. P.; Yang, C. C.; Karuppiah, C. $SrMnO_3$/Functionalized h-BN Composite Modified Disposable Sensor for the Voltammetric Determination of Furaltadone Antibiotic Drug. *Catalysts* **2022**, 12, 1494−1511.

(7) Mariappan, K.; Chen, T. W.; Chen, S. M.; Tseng, T. W.; Bian, Y.; Sun, T. T.; Jiang, J.; Yu, J. Fabrication of Hexagonal $CuCoO_2$ Modified Screen-Printed Carbon Electrode for the Selective Electrochemical Detection of Furaltadone. *Int. J. Electrochem. Sci* **2022**, 17, 220644−220659.

(8) Vasu, D.; Karthi Keyan, A.; Sakthinathan, S.; Chiu, T. W. Investigation of electrocatalytic and photocatalytic ability of Cu/Ni/TiO_2/MWCNTs Nanocomposites for detection and degradation of antibiotic drug Furaltadone. *Scientific Reports* **2022,** 12, 886−892.

(9) Amalraj, A. J. J.; Murthy, U. N.; Sea-Fue, W. Ultrasensitive electrochemical detection of an antibiotic drug furaltadone in fish tissue with a ZnO-$ZnCo_2O_4$ self-assembled nano-heterostructure as an electrode material. *Microchemical Journal* **2021**, 169, 106566−106575.

(10) Balamurugan, K.; Rajakumaran, R.; Chen, S. M.; Karthik, R.; Shim, J. J.; Shafi, P. M. Massive engineering of spinel cobalt tin oxide/tin oxide-based electrocatalyst for the selective voltammetric determination of antibiotic drug furaltadone in water samples *Journal of Alloys and Compounds.* **2021**, 15, 160750−160761.

(11) Sheng, L. Q.; Chen, M. M.; Chen, S. S.; Du, N. N.; Liu, Z. D.; Song, C. F.; Qiao, R. High-performance liquid chromatography with fluorescence detection for the determination of

nitrofuran metabolites in pork muscle. *Food Additives & Contaminants: Part A* **2013,** 30, 2114−2122.

(12) Zhang, S.; Jin, M. L.; Gao, Y. X.; Li, W. Q.; Wang, X. Y.; Li, X. X.; Qiao, J. Q.; Peng, Y. Histidine-capped fluorescent copper nanoclusters: an efficient sensor for determination of furaltadone in aqueous solution. *Chemical Papers*, **2022**, 76, 7855−7863.

(13) Geng, J.; Sun, J.; Lin, H.; Wang, X. Metal-regulated d 10 coordination polymers constructed from bis (pyridyl)-bis (amide) ligands with different spacers as high-efficiency fluorescence sensors for identifying chlortetracycline and furaltadone. *New Journal of Chemistry*, **2023**, 47, 9701−9707.

(14) Cai, Z.; Li, H.; Yang, X.; Zhang, M.; Guo, J.; Su, Y.; Liu, T. Blue-emitting tryptophan-protected gold nanoclusters acted as a sensitive nanosensor for fluorescence sensing and visual imaging detection of furaltadone. *Spectrochim. Acta A Mol. Biomol. Spectrosc.*, 308 (2024) 123748.

(15) For PA@AuNPs characterization refer to A. Sowndarya, T. Daniel Thangadurai, D. Nataraj, Morphology-transforming AuNPs-based Fluorescent probe for Ultra-low sensitive detection of Levofloxacin in Urine samples at pH 7.0 through Excimer formation, *J. Mol. Liquids* 407 (2024) 125156.
